# Human resource needs and costs for HIV pre-exposure prophylaxis provision in nurse-led primary care in Eswatini and opportunities for task sharing﻿

**DOI:** 10.1186/s12960-022-00770-9

**Published:** 2022-10-23

**Authors:** Stefan Kohler, Shona Dalal, Anita Hettema, Sindy Matse, Till Bärnighausen, Nicolas Paul

**Affiliations:** 1 Heidelberg Institute of Global Health, Medical Faculty and University Hospital, Heidelberg University, Heidelberg, Germany; 2 Institute for Social Medicine, Epidemiology and Health Economics, Charité – Universitätsmedizin Berlin, corporate member of Freie Universität Berlin and Humboldt Universität zu Berlin, Berlin, Germany; 3 Department of Global HIV, Hepatitis and Sexually Transmitted Infections Programmes, World Health Organization, Geneva, Switzerland; 4 Clinton Health Access Initiative, Mbabane, Eswatini; 5 Eswatini Ministry of Health, Mbabane, Eswatini

**Keywords:** Costs and cost analysis, Health personnel, HIV pre-exposure prophylaxis, Nurse-led primary health care, Sub-Saharan Africa, Task performance and analysis, Task sharing, Task shifting, Time-and-motion study

## Abstract

**Background:**

The global expansion of HIV pre-exposure prophylaxis (PrEP) includes health systems that face a shortage of skilled health care workers (HCWs). We estimated the human resource needs and costs for providing PrEP in nurse-led primary care clinics in Eswatini. Furthermore, we assessed potential cost savings from task sharing between nurses and other HCW cadres.

**Methods:**

We conducted a time-and-motion and costing study in a PrEP demonstration project between August 2017 and January 2019. A form for recording time and performed activities (“motion”) was filled by HCWs of six primary care clinics. To estimate the human resource needs for specific PrEP activities, we allocated recorded times to performed PrEP activities using linear regression with and without adjusting for a workflow interruption, that is, if a client was seen by different HCWs or by the same HCW at different times. We assessed a base case in which a nurse provides all PrEP activities and five task shifting scenarios, of which four include workflow interruptions due to task sharing between different HCW cadres.

**Results:**

On average, PrEP initiation required 29 min (95% CI 25–32) of HCW time and PrEP follow-up 16 min (95% CI 14–18). The HCW time cost $4.55 (uncertainty interval [UI] 1.52–9.69) for PrEP initiation and $2.54 (UI 1.07–4.64) for PrEP follow-up when all activities were performed by a nurse. Time costs were $2.30–4.25 (UI 0.62–9.19) for PrEP initiation and $1.06–2.60 (UI 0.30–5.44) for PrEP follow-up when nurses shared tasks with HCWs from lower cadres. Interruptions of the workflow added, on average, 3.4 min (95% CI 0.69–6.0) to the time HCWs needed for a given number of PrEP activities. The cost of an interrupted workflow was estimated at $0.048–0.87 (UI 0.0098–1.63) depending on whose time need increased.

**Conclusions:**

A global shortage of skilled HCWs could slow the expansion of PrEP. Task shifting to lower-cadre HCW in nurse-led PrEP provision can free up nurse time and reduce the cost of PrEP provision even if interruptions associated with task sharing increase the overall human resource need.

**Supplementary Information:**

The online version contains supplementary material available at 10.1186/s12960-022-00770-9.

## Introduction

Pre-exposure prophylaxis (PrEP) can effectively reduce the risk of acquiring HIV [1]. The World Health Organization (WHO) recommends the provision of PrEP to people at substantial risk of HIV infection since 2015 [2]. After initial PrEP demonstration projects had been implemented, Eswatini and other high HIV-burden countries began expanding the provision of oral PrEP [3]. In Eswatini, PrEP has been added to existing HIV prevention services and is presently provided through nurse-led primary care clinics in the public health care system and through private clinics.

Shortages of qualified staff have been described as a barrier to PrEP implementation in Sub-Saharan Africa [4–6]. Information about the human resource needs and costs for PrEP provision can help identify and close potential staffing and financing gaps. Time-and-motion studies are a tool to assess human resource needs and optimize work processes [7]. We conducted a time-and-motion study to observe and record how health care workers (HCWs) spent time on the provision of PrEP activities during a PrEP demonstration project in Eswatini. Furthermore, we valued the HCW time needed for PrEP care and simulated different task shifting scenarios between nurses and other HCW cadres. Nurses and doctors are presently the main providers of PrEP care [8, 9], and shifting tasks from them to other HCW cadres could facilitate the global scale-up of PrEP. Task sharing has been used in previous efforts to scale-up access to HIV treatment and other chronic disease care [10–12].

Others have conducted time-and-motion studies of PrEP care in different contexts and with smaller study samples [13–21]. The reported median times for PrEP initiation varied from 18 min (IQR 15–26) [20] to 51 min (IQR 43–63) [19]. Median times for PrEP follow-up varied from 8.5 min (IQR 7.75–9.75) [18] to 33 min (IQR 24–35) [13] (Additional file 1: Supplement A Table S1). We complement previous studies by estimating the human resource needs for the provision of PrEP through primary care clinics in Eswatini. We also add to the existing literature by assessing the cost impact of shifting PrEP care activities from nurses to lower cadre HCWs (e.g., nurse assistants, HIV testing services counselors, or peer supporters).

The aims of this study were, first, to assess the time HCWs spent on PrEP activities in a demonstration project providing PrEP through nurse-led primary care clinics in Eswatini; second, to assess the financial costs of HCW time spent on PrEP activities to the Eswatini health system; and third, to assess potential cost savings from shifting PrEP tasks from nurses to lower-cadre HCWs.

## Methods

### Study setting

Eswatini shares borders with Mozambique and South Africa. It has been among the first countries to implement PrEP care for all people at substantial risk of HIV infection [3]. The HIV prevalence among adults aged 15–49 years was estimated at 36.1% for women and 19.4% for men in 2021 [22]. A nationwide PrEP scale-up began in 2019 after the provision of PrEP through nurse-led primary care clinics had been piloted in demonstration projects in the Hhohho, Manzini, and Shiselweni regions of Eswatini. While PrEP is being scaled-up, the number of nurses has decreased from 41 to 25 per 10,000 population between 2018 and 2020 [23].

The study at hand has been a part of the 18-month PrEP demonstration project in the Hhohho region, which has been conducted between August 1, 2017, and January 31, 2019 [24]. In 2017, 29% of Eswatini’s 1.1 million people lived in the northwestern Hhohho region [25]. Six government primary care clinics that served about 20% of the Hhohho population were purposively selected for the PrEP demonstration project. These clinics were nurse-led middle- to high-volume government health facilities and provided HIV services free of charge. Prior to the demonstration project, no clinic had experience with PrEP care. Six months into the demonstration project, clinics gradually introduced a PrEP promotion package as part of a stepped-wedge randomized trial (Additional file 1: Supplement A Table S2).

### PrEP provision in primary care clinics

During a PrEP initiation visit, clinic clients interacted with one or more HCWs for pre-PrEP counseling, HIV risk assessment, blood testing, PrEP eligibility screening, and eventually PrEP initiation if eligible for and interested in PrEP (see PrEP demonstration project standard operating procedure and guidelines; Additional file 2: Supplement B, Additional file 3: Supplement C [26, 27]). Clients proceeding to PrEP initiation were supplied with 1 month of PrEP drugs. PrEP follow-ups were scheduled approximately 1 month and 3 months after initiation and then 3-monthly. Regular PrEP follow-up visits included follow-up counseling, blood testing, and a PrEP drug refill. Other follow-up visits included PrEP restarts, side-effect counseling and treatment, or PrEP cessation. Blood testing during PrEP care included taking a finger-prick blood sample for point-of-care HIV and hepatitis tests and taking a venous blood sample for creatinine testing in the regional government laboratory [28]. The type of PrEP visit and test availability determined which blood tests were conducted. Of 2232 clinic attendees which were approached for an HIV risk assessment during the 18-month demonstration project, 2168 underwent a risk assessment, 1538 were identified as being at risk, and 517 initiated PrEP. Most people initiating PrEP attended the clinic for reasons other than PrEP. Of the 504 clinic clients who started PrEP at least month before the end of the demonstration project, 324 returned for at least one PrEP follow-up visit [24].

### Health care worker cadres involved in PrEP care

The standard operating procedure and guidelines of the PrEP demonstration project recommended that a nurse or higher qualified HCW conducts PrEP eligibility screening and PrEP initiation. PrEP initiation in addition required training in nurse-led antiretroviral therapy initiation in the Hhohho region of Eswatini (NARTIS). Pre-PrEP counseling, HIV risk-assessment, and parts of the eligibility screening could also be conducted by an HTS counselor. Pre-PrEP counseling and HIV risk-assessment could also be provided by a peer supporter, like a mother-to-mother mentor or an expert client (i.e., a long-term adherent antiretroviral therapy user). Blood testing was not specifically regulated for PrEP care. In practice, HCWs other than nurses were only present at some clinics, and PrEP activities were also provided by nurse assistants (Additional file 1: Supplement A Tables S3–S4).

### Study design

We conducted a time-and-motion and costing study to assess the human resource needs and costs for PrEP provision. Time-and-motion data was collected at the six demonstration project clinics throughout the PrEP demonstration project. Weekdays from Monday to Thursday were randomly pre-scheduled as study days. The actual data collection was subject to the availability of a research assistant to travel from the study office in Mbabane to a clinic to hand out and collect time-and-motion forms. The exact times of the day when the time-and-motion study was conducted were variable due to different travel distances and traffic conditions. The costing study valued the human resource needs from the time-and-motion study with government salaries. To emphasize routine PrEP care after training and initial learning, we excluded time-and-motion data from the first month of the demonstration project and from study mentors. As no time-and-motion was collected in the second month of the demonstration project, the study assessed PrEP care after HCWs had two or more months of experience with PrEP care.

### Data collection and processing

For clinic attendees expressing interest in PrEP, a form for recording time and conducted activities (“motion”) was attached to the client’s paper file (Additional file 4: Supplement D). HCWs were asked to document when an interaction with a client started and ended, which type of PrEP activities were conducted during their interaction, and how much time of the interaction was used for non-PrEP activities. PrEP activities could be marked as pre-PrEP counseling, HIV risk assessment, PrEP eligibility screening, PrEP initiation, PrEP follow-up, treatment of PrEP side-effects, and laboratory tests for PrEP. Filled time-and-motion forms were collected by the research assistant before leaving the clinic on a time-and-motion study day and entered in a spreadsheet. Few clients had the same PrEP activity recorded in two separate time-and-motion forms during one clinic visit, either because one HCW interrupted an activity or because two HCWs were included in the same PrEP activity. These time-and-motion data were consolidated into one record with the total HCW time spent on the same PrEP activity. Summary statistics of the collected time-and-motion data and HCW involvement in PrEP provision were calculated using the data as recorded. All other data analysis was based on the consolidated data.

### Data analysis

The time a HCW spent on PrEP care was calculated by subtracting the time recorded for non-PrEP care from the total time recorded for an interaction of a HCW with a clinic attendee. Summary statistics of the time need for PrEP care were calculated for the sequences of PrEP activities recorded during a clinic visit. Clinic visits in which clinic attendees had their first contact with PrEP were classified as PrEP initiation visits, irrespective of whether a clinic attendee proceeded to PrEP initiation. PrEP initiation visits included up to five PrEP activities (i.e., pre-PrEP counseling, HIV risk assessment, blood testing, PrEP eligibility screening, and PrEP initiation). Other clinic visits with PrEP activities were classified as PrEP follow-up visits and included up to two PrEP activities (i.e., follow-up counseling and blood testing).

We used two linear regression models to decompose the time that HCWs recorded for sequences of PrEP activities into time needed for specific PrEP activities with and without adjusting for an interrupted workflow. The regression models for these analyses were selected from a broader set of models and had the smallest Bayesian information criterion in comparison to alternative model specifications (Additional file 1: Supplement A Table S5). The first regression model includes only PrEP activities and a constant term (i.e., a fixed time cost) as independent variables. It was used to estimate an average time need for each PrEP activity and to predict an average duration of a PrEP initiation visit and a PrEP follow-up visit. The second regression model includes the number of provided PrEP activities and whether a workflow was interrupted as covariates. It was used to analyze the characteristics of the process in which PrEP activities were provided. The PrEP care workflow was considered as interrupted if a client was seen either by different HCWs or by the same HCW at different times.

To assess the financial human resource costs from a health system perspective, we multiplied the estimated time needs for PrEP activities with government salaries. The main analysis estimated average costs throughout the demonstration project based on the first regression model. Task shifting scenarios were analyzed using the estimates of the second regression model. HCWs costs at the average salary level of a HCW cadre in 2018 were used, and benefits but no overhead costs were added. Costs were converted from Eswatini Lilangeni (SZL) to United States dollar ($) using the annual exchange rate for 2018 of $1 = SZL13.234 [29]. HCW costs per minute were calculated assuming 220 workdays per year and 8 workhours per day. In the main analysis and costing base case, all estimated time needs were valued with the cost of nurse time. Nurses were qualified to provide all PrEP activities and available throughout primary care clinics in Eswatini. In task shifting scenarios, the estimated time needs were valued with the cost of nurses and other HCW cadres. Five scenarios were assessed. Scenarios 1–3 represent practiced task shifting and task sharing. Scenarios 4 and 5 represent additional opportunities for task sharing. All analyses were conducted in Stata 15.1 SE.

### Uncertainty analyses

For time needs that were estimated through linear regression, 95% confidence intervals (CIs) were reported. To allow for uncertainty of the estimated time needs and salary structure of a specific clinic, uncertainty intervals (UIs) were calculated. The UIs were calculated by multiplying the upper bound of the 95% CI for the HCW time needed with the highest salary level of a HCW cadre and the lower bound with the lowest salary level of a HCW cadre. If the lower bound was negative, the lower cost bound was set to zero. In tasks sharing scenarios with two or more HCW cadres, the fixed time cost was valued with the lowest as well as the highest salary of the involved HCW cadres and reported as a cost range. UIs for cost sums and differences were estimated by calculating the minimum and maximum values possible within the UI bounds of the added or subtracted cost estimates.

## Results

### Sample characteristics

After excluding 19 time-and-motion forms from the first month of PrEP care or from study mentors, we evaluated 304 time-and-motion forms filled out by 34 HCWs between October 3, 2017, and January 30, 2019. The 601 PrEP activities recorded during this period were provided to 190 clinic attendees during 213 clinic visits. 186 time-and-motion forms were filled out during 130 PrEP initiation visits and 118 forms during 83 PrEP follow-up visits. The HCWs recording time and motion were 3 nursing sisters, 19 registered nurses, 3 nurse assistants, 6 HIV testing services (HTS) counselors, 2 phlebotomists, 1 mother-to-mother mentor, and no expert client. In total, the time-and-motion recording captured 4583 min (9.5 working days) of HCW time spent on PrEP activities.

Clinic visits with PrEP activities had a median of 2 (IQR 1–2) PrEP-related client–HCW interactions with 1 HCW (IQR 1–2). The median PrEP initiation visit included 3 (IQR 2–3) PrEP activities. The median PrEP follow-up visit included 1 (IQR 1–2) PrEP activity. Nurses and nursing sisters were involved in 41% (247) of the 601 PrEP activities recorded, nurse assistants in 9.0% (54), and lay HCWs (mother-to-mother mentors) in 0.33% (2). HTS counselors and phlebotomists were only available at some clinics. They provided 48% (289) and 1.5% (9) of the recorded PrEP activities, respectively. Nurses and nursing sisters engaged most frequently in PrEP initiation (24% [59] of 247 PrEP activities provided by nurses), eligibility screening (23% [57]) and follow-up counseling (22% [55]). HTS counselors engaged most frequently in blood testing (28% [80] of 289 PrEP activities provided by HTS counselors), pre-PrEP counseling (27% [78]), and HIV risk assessment (26% [75]). Pre-PrEP counseling and HIV risk assessment were the only PrEP activities provided by mother-to-mother mentors (100% [2] of 2 PrEP activities provided by peer supporters). No expert client participated in the time-and-motion study (Additional file 1: Supplement A Table S4).

### Human resource needs

Adding-up the time of similar PrEP activities that were recorded in more than one time-and-motion form for the same client resulted in 284 sequences with 574 unique PrEP activities. Single PrEP activities required between 3 and 36 min worktime from a HCW and sequences of PrEP activities up to 42 min. HCWs recorded a median time of 31 min (IQR 20–37) for the sequence of PrEP activities leading to PrEP initiation (i.e., pre-PrEP counseling, HIV risk assessment, blood testing, PrEP eligibility screening, and PrEP initiation). For pre-PrEP counseling and HIV risk assessment only, a median worktime of 11 min (IQR 10–14) was recorded. Regular PrEP follow-up visits with follow-up counseling and blood testing required a median worktime of 15 min (IQR 10–19). Related to the HCW qualification requirements, some PrEP activities were commonly provided together in a sequence of PrEP activities. Examples of common sequences of PrEP activities include pre-PrEP counseling, HIV risk assessment and blood testing, PrEP eligibility screening and initiation, or follow-up counseling and blood testing. An interrupted workflow occurred in 56% (160 of 284) of the PrEP activity sequences. The workflow was always interrupted when a client interacted with more HCWs and rarely when a client interacted with only one HCW during a PrEP clinic visit (100% [156 of 156] versus 3.1% [4 of 128] of the respective PrEP activity sequences; Pearson χ^2^ test *P* < 0.001) (Table 1).

**Table 1:** Health care worker time recorded for sequences of PrEP activities in a time-and-motion study in six nurse-led primary care clinics in Eswatini, 2017–2019

Health care worker time for sequences of PrEP activities (minutes)	*N*	Mean (range)	Median (IQR)
PrEP initiation visit			
Pre-PrEP counseling	2	9 (8–10)	9 (8–10)
HIV risk assessment	2	11.5 (6–17)	11.5 (6–17)
PrEP eligibility screening	1	30	30
PrEP initiation	7	24.9 (19–31)	24 (20–30)
Pre-PrEP counseling/HIV risk assessment	42	12.2 (5–24)	11 (10–14)
Pre-PrEP counseling/–/blood testing	1	23	23
Pre-PrEP counseling/–/–/–/PrEP initiation	1	20	20
–/–/–/PrEP eligibility screening/PrEP initiation	41	19.4 (8–31)	20 (15–23)
Pre-PrEP counseling/HIV risk assessment/blood testing	40	16.8 (4–30)	15 (12–21)
Pre-PrEP counseling/HIV risk assessment/–/PrEP eligibility screening	18	18.1 (5–29)	17.5 (15–23)
–/–/blood testing/PrEP eligibility screening/PrEP initiation	1	16	16
Pre-PrEP counseling/HIV risk assessment/blood testing/PrEP eligibility screening	2	23.5 (15–32)	23.5 (15–32)
Pre-PrEP counseling/HIV risk assessment/–/PrEP eligibility screening/PrEP initiation	6	28.3 (19–37)	28 (23–35)
Pre-PrEP counseling/HIV risk assessment/blood testing/PrEP eligibility screening/PrEP initiation	9	29 (13–42)	31 (20–37)
PrEP follow-up visit			
Follow-up counseling	56	13.1 (3–36)	11 (8–15)
Follow-up counseling/blood testing	27	15.7 (5–37)	15 (10–19)
PrEP blood testing			
Blood testing	28	11.9 (3–30)	10 (7.5–15)

*N* = 284. IQR = interquartile range. /–/ = a PrEP activity that would be expected in a sequence of PrEP initiation or follow-up activities was not recorded in the time-and-motion data of a clinic attendee. A PrEP initiation visit resulting in PrEP initiation includes pre-PrEP counseling, HIV risk assessment, blood testing, PrEP eligibility screening, and PrEP initiation. Regular PrEP follow-up visits include follow-up counseling, blood testing, and drug refill

**Fig. 1:**
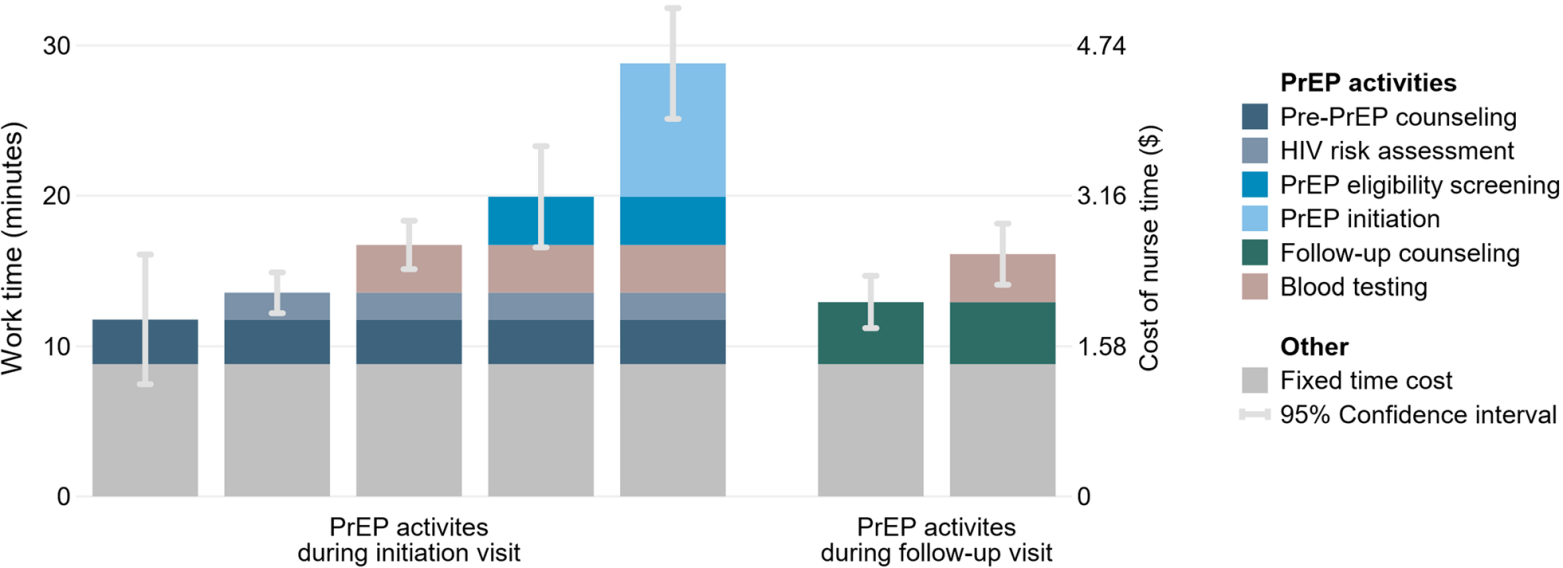
Human resource needs and costs for the provision of PrEP in primary care clinics in Eswatini. *N* = 284

The regression decomposition of the recorded sequences of PrEP activities without covariates estimated a fixed time cost of 8.8 min (95% CI 6.1–12) and allocated additional 3.0 min (95% CI − 1.3–7.2) to pre-PrEP counseling, 1.8 min (95% CI − 2.6–6.1) to HIV risk-assessment, 3.2 min (95% CI 0.0042–6.4) to PrEP eligibility screening, 8.9 min (95% CI 4.8–13) to PrEP initiation, and 4.1 min (95% CI 1.2–7.0) to PrEP follow-up counseling. A time need of 3.2 min (95% CI 1.3–5.0) was estimated for blood testing. Summing-up the fixed time cost and time need for single PrEP activities, a PrEP initiation visit was estimated to require 29 min (95% CI 25–32) of HCW time. A PrEP follow-up visit with blood testing was estimated to require 16 min (95% CI 14–18) of HCW time (Table 2 Model 1, Fig. 1).

**Table 2:** Estimated and predicted health care worker time for providing PrEP in nurse-led primary care clinics in Eswatini

Health care worker time	Model 1 Coef. (95% CI)	Model 2 Coef. (95% CI)
PrEP activity (estimated minutes)		
Pre-PrEP counseling	3.0 (− 1.3–7.2)	4.3 (− 0.14–8.7)
HIV risk assessment	1.8 (− 2.6–6.1)	3.3 (− 1.4–7.9)
PrEP eligibility screening	3.2 (0.0042–6.4)^*^	3.8 (0.37–7.2)^*^
PrEP initiation	8.9 (4.8–13)^***^	11 (6.9–16)^***^
Follow-up counseling	4.1 (1.2–7.0)^**^	4.7 (1.7–7.7)^**^
Blood testing	3.2 (1.3–5.0)^***^	4.0 (1.8–6.2)^***^
Covariates (estimated minutes)		
Number of provided PrEP activities		− 1.5 (− 2.7 to − 0.30)^*^
Interrupted workflow		3.4 (0.69–6.0)^*^
Fixed time cost (estimated minutes)		
Constant	8.8 (6.1–12)^***^	8.9 (5.8–12)^***^
PrEP clinic visit (predicted minutes)		
PrEP initiation visit	29 (25–32)^***^	28–32 (25–36)^***,†^
PrEP follow-up visit	29 (25–32)^***^	15–18 (12–21)^***,†^
Model statistics		
Adjusted *R*^2^	0.24	0.26
AIC/BIC	1894/1920	1891/1924

*N* = 284. ^*^P < 0.05, **P < 0.01, ***P < 0.001. ^†^Range represents prediction without and with an interrupted workflow. PrEP activities add time to the fixed time cost

In the regression model with covariates, conducting more PrEP activities reduced the total HCW time need by 1.5 min (95% CI 0.30–2.7) per activity, and a workflow interruption added 3.4 min (95% CI 0.69–6.0) to the total HCW time need (Table 2 Model 2). These estimates remained similar when additional covariates were added to the regression model. An additional month of experience with PrEP provision reduced the HCW time need by 0.37 min (95% CI 0.11–0.63). There was no evidence for an additional association of the HCW time need and the introduction of a PrEP promotion package or the involvement of a nurse in PrEP provision (Additional file 1: Supplement A Table S5 Model 5). Whether a workflow was interrupted differed by clinic (Pearson χ^2^ test *P* < 0.001) and ceased to be associated with the HCW time need after additionally adjusting for clinic specific time needs. Clinic fixed effects were individually but not jointly significant (Additional file 1: Supplement A Table S5 Model 6).

### Human resource costs

Depending on qualification and experience level of the HCWs, the health system costs of human resources varied between $0.014 per minute for peer supporters and $0.26 (range 0.24–0.27) per minute for a nursing sister. The cost for nurse time was $0.16 per minute (range 0.12–0.20). Valuing the estimated time per PrEP activity with the cost of a nurse of $0.16 (range 0.12–0.20) per minute and without adjusting for an interrupted workflow, we estimated a fixed time cost of $1.39 (UI 0.76–2.27) and additional costs of $0.47 (UI 0–1.42) for pre-PrEP counseling, $0.28 (UI 0–1.21) for HIV risk assessment, $0.51 (UI 0.00052–1.26) for PrEP eligibility screening, $1.40 (UI 0.60–2.55) for PrEP initiation, $0.65 (UI 0.15–1.38) for follow-up counseling, and $0.50 (UI 0.16–0.99) for PrEP-related blood testing. The fixed time cost ranged from $0.12 (UI 0.086–0.16) for a peer supporter to $2.26 (UI 1.48–3.13) for a nursing sister. The additional cost of pre-PrEP counseling ranged from $0.042 (UI 0–0.10) for the health system if provided by a peer supporter to $0.76 (UI 0–1.95) if provided by a nursing sister. The estimated cost of follow-up counseling varied between $0.19 (UI 0.058–0.33) if provided by an HTS counselor and $1.06 (UI 0.30–1.91) if provided by a nursing sister (Table 3).

**Table 3:** Predicted human resource costs for PrEP activities by health care worker qualification

Human resource costs ($)	Nursing sister	Nurse	Nurse assistant	Phlebotomist	HTS counselor	Peer supporter
Unit cost of time						
Salary per minute	0.26 (0.24–0.27)	0.16 (0.12–0.20)	0.11 (0.11–0.12)	0.063 (0.055–0.072)	0.047	0.014
Fixed time cost						
Fixed cost per PrEP visit	2.26 [1.48–3.13]	1.39 [0.76–2.27]	0.99 [0.66–1.36]	0.56 [0.34–0.83]	0.42 [0.29–0.54]	0.12 [0.086–0.16]
Cost of PrEP activity						
Pre-PrEP counseling	0.76 [0–1.95]	0.47 [0–1.42]	0.33 [0–0.85]		0.14 [0–0.34]	0.042 [0–0.10]
HIV risk assessment	0.45 [0–1.66]	0.28 [0–1.21]	0.20 [0–0.72]		0.083 [0–0.29]	0.025 [0–0.087]
PrEP eligibility screening	0.82 [0.0010–1.74]	0.51 [0.00052–1.26]	0.36 [0.00045–0.76]			
PrEP initiation	2.28 [1.17–3.51]	1.40 [0.60–2.55]	1.00 [0.52–1.53]			
Follow-up counseling	1.06 [0.30–1.91]	0.65 [0.15–1.38]	0.46 [0.13–0.83]		0.19 [0.058–0.33]	
Blood testing	0.82 [0.32–1.37]	0.50 [0.16–0.99]	0.36 [0.14–0.59]	0.20 [0.072–0.36]	0.15 [0.062–0.24]	

() = range. [] = uncertainty interval. Nurse = registered nurse with single or double qualification. HTS = HIV testing services. Peer supporter = mother-to-mother mentor or expert client. Empty cell = health care worker unlikely to provide this PrEP activity. Time needs estimated based on Table 2 Model 1. Time needs estimated based on Table 2 Model 2 are provided in Additional file 1: Supplement A Table S6

By adding-up the human resource costs of the involved PrEP activities, we estimated a human resource cost of $2.25–7.40 (UI 0.75–13) for a PrEP initiation visit and $1.03–4.14 (UI 0.48–6.41) for a PrEP follow-up visit. We estimated a cost of $4.55 (UI 1.52–9.69) for a PrEP initiation visit and $2.54 (UI 1.07–4.64) for a PrEP follow-up visit if all PrEP activities are provided a nurse (Fig. 1, Additional file 1: Supplement A Table S6).

### Task sharing and cost savings

If all PrEP activities are provided a nurse and the workflow is not interrupted, we estimated a cost of $4.47 (UI 1.24–11) for a PrEP initiation visit and $2.31 (UI 0.71–4.98) for a PrEP follow-up visit (Table 4 Base case). Human resource costs are higher if all PrEP activities are provided by a higher HCW cadre like a nursing sister (Scenario 1). The cost of a PrEP initiation visit decreases to $2.85–4.25 (UI 0.83–9.19) if pre-PrEP counseling, HIV risk assessment, and blood testing are shifted to an HTS counselor and a phlebotomist or to an HTS counselor only (Scenarios 2 and 3). PrEP initiation cost decrease further to $2.30–4.21 (UI 0.62–9.04) if, in addition, pre-PrEP counseling and HIV risk assessment are shifted to a mother-to-mother mentor or an expert client (Scenarios 4 and 5). The human resource cost for a PrEP follow-up visit are $1.20–2.60 (UI 0.30–5.44) if a nurse shares PrEP follow-up activities with an HTS counselor or a phlebotomist (Scenarios 2 and 3). The human resource cost for a PrEP follow-up visit decrease further to $1.06–1.86 (UI 0.30–3.28) if follow-up counseling is provided by a nurse assistant and blood testing is conducted by an HTS counselor instead of a nurse (Scenarios 4 and 5).

**Table 4:** Predicted human resource costs for a PrEP initiation and a PrEP follow-up visit with task shifting and sharing

Scenario	Pre-PrEP counseling	HIV risk assessment	PrEP eligibility screening	PrEP initiation	Follow-up counseling	Blood testing	PrEP initiation visit ($)^#^	PrEP follow-up visit ($)^#^
Base case^†^	Nurse	Nurse	Nurse	Nurse	Nurse	Nurse	4.47 [1.24–11]	2.31 [0.71–4.98]
Scenario 1^†^	Nursing sister	Nursing sister	Nursing sister	Nursing sister	Nursing sister	Nursing sister	7.27 [2.43–15]	3.76 [1.40–6.87]
Scenario 2^‡^	HTS counselor	HTS counselor	Nurse	Nurse	Nurse	Phlebotomist	2.89–4.25 [0.83–9.19]	1.44–2.60 [0.35–5.44]
Scenario 3^‡^	HTS counselor	HTS counselor	Nurse	Nurse	Nurse	HTS counselor	2.85–4.21 [0.83–9.04]	1.20–2.56 [0.30–5.29]
Scenario 4^‡^	HTS counselor	HTS counselor	Nurse	Nurse	Nurse assistant	HTS counselor	2.85–4.21 [0.83–9.04]	1.06–1.86 [0.30–3.28]
Scenario 5^‡^	Peer supporter	Peer supporter	Nurse	Nurse	Nurse assistant	HTS counselor	2.30–4.06 [0.62–8.51]	1.06–1.86 [0.30–3.28]

[] = uncertainty interval. HCW = health care worker. HTS = HIV testing services. Peer supporter = mother-to-mother mentor or expert client. ^†^Uninterrupted workflow possible when tasks are fully shifted between HCW cadres. ^‡^Interrupted workflow when tasks are shared among HCW cadres. ^#^Cost range with fixed time cost based on lowest and highest HCW cadre salary when different HCW cadres provide PrEP activities. Scenarios 1–3 represent practiced task shifting and task sharing. Scenarios 4 and 5 represent additional opportunities for task sharing. Time needs estimated based on Table 2 Model 2. Time needs estimated based on Table 2 Model 1 are provided in Additional file 1: Supplement A Table S7

At average salary levels, task sharing between nurses and HCWs of a lower cadre decreased the human resource cost of a PrEP initiation visit in the assessed scenarios. Sharing PrEP follow-up activities between a nurse and other HCW cadres decreased or increased personnel costs. If all PrEP follow-up activities are shifted from nurses to lower-cadre HCWs, then costs are saved even if lower-cadre HCWs share tasks. Task sharing between HCWs is only possible if a sequence of PrEP activities is interrupted. We estimated that an interrupted workflow adds on average 3.4 min (95% CI 0.69–6.0) to the HCW time needed for an otherwise similar sequence of PrEP activities. Valuing this additional time need with the lowest and highest HCW time cost suggests an additional cost of $0.048–0.87 (UI 0.0098–1.64) if task sharing or other reasons interrupt a previously uninterrupted workflow. Depending on how HCW cadres share the cost of an interrupted workflow and the fixed time cost, potential cost savings from shifting PrEP activities from nurses to lower HCW cadres were $0.22–2.17 (UI − 7.94–11) for a PrEP initiation visit and $− 0.29–1.25 (UI − 4.72–4.68) for a PrEP follow-up visit (Base case, Scenarios 2–5). In terms of potentially freed up nurse time, task sharing could shift 3.8–16 min (CI -0.69–18) and − 2.3–15 min (CI − 6.6–17) worktime from nurses to other HCW cadres for each PrEP initiation and follow-up visit, respectively (predicted based on Table 2 Model 2).

## Discussion

### Summary of findings

We conducted a time-and-motion study and valued time with HCW salaries to assess the human resource needs and costs for PrEP provision in nurse-led primary care in Eswatini. In addition, we assessed the cost impact of sharing PrEP tasks between nurses and other HCW cadres. Our analyses assessed oral PrEP care in a demonstration project after initial training and learning. An average PrEP initiation visit was estimated to have required 29 min (95% CI 25–32) HCW time. An average PrEP follow-up visit with blood testing was estimated to have required 16 min (95% CI 14–18) HCW time. When all PrEP activities were provided by a nurse, this HCW time cost the health system $4.55 (UI 1.52–9.69) and $2.54 (UI 1.07–4.64), respectively. Task sharing between nurses and HCW with less training reduced the HCW time cost to as low as $2.30–4.06 (UI 0.62, 8.51) for a PrEP initiation visit and $1.06–1.86 (UI 0.30, 3.28) for a PrEP follow-up visit. Sharing tasks between different HCW cadres introduced interruptions to the workflow. Interrupted workflows were estimated to have added, on average, 3.4 min (95% CI 0.69–6.0) or a cost of $0.048–0.87 (UI 0.0098–1.64) in comparison to an otherwise uninterrupted workflow.

### Findings in context

Time-and-motion studies have been previously applied to assess the human resource needs for providing PrEP, for instance, to HIV-discordant couples in Kenya, Uganda, and Zimbabwe [13–16]; to adolescent girls and young women in Kenya and South Africa [17–19]; to women attending ante- or postnatal care in Kenya [20]; or to men who have sex with men in the US [21]. The recorded duration for PrEP initiation ranged from a median time of 13.6 min to a mean time of 51 min. The median time reported for individual PrEP follow-up ranged from 8.5 min to 46 min (Additional file 1: Supplement A Table S1).

Our estimated HCW time needs are in the lower range of previously reported values. Differences in recorded time needs may partially be explained by PrEP provision through more specialized health care providers [13, 16], by the focus on other PrEP target groups, by the integration of PrEP care in other health services [20], or by the level of experience that providers had with PrEP care. This study also complements other findings from the same PrEP demonstration project. In a study of the out-of-pocket expenses and time spent on clinic visits, we found that PrEP clients’ median time spent in a clinic was 2.53 h (IQR 1.55–3.43) for PrEP initiation and 1.89 h (IQR 0.88–2.75) for PrEP follow-up [30]. The discrepancy between the HCW time need for PrEP care activities and the time that PrEP clients spent in a clinic suggests substantial waiting time. A qualitative study with HCWs that were purposively selected for in-depth interviews within the demonstration project reported that PrEP clients and HCWs perceived the PrEP initiation process as too long [31].

We are not aware of other studies that have quantified the implications of task sharing and shifting in PrEP care. Task shifting has been recommended by the WHO as an approach to react to a scarcity of qualified HCW [32, 33]. Human resources for health have been considered particularly scarce in Africa and in HIV services [34, 35]. A systematic review on low-income and middle-income countries found that task shifting may reduce costs and enhance efficiency in HIV care [36]. Another systematic review concluded that shifting the provision of antiretroviral therapy from doctors to trained lower-cadre HCW in Africa was possible without decreasing the quality of care [10]. Task shifting between nurses and HCW cadres has been discussed for PrEP care [5, 37]. In a qualitative study in Canada, community health workers appeared open to provide PrEP services [38].

### Practical implications

Our findings on the human resource needs and costs for primary care-based PrEP provision can guide human resource planning in efforts to expand PrEP to all people at substantial risk of HIV infection in Eswatini and comparable care settings. Our analysis of where and how much HCW time is needed in PrEP care may inform approaches to simplify and shorten clinic visits for PrEP. The time needs and variability in time need described in this study can inform discussions about opportunities for process optimization (e.g., through task sharing, minimizing fixed time costs, or reducing workflow interruptions). Furthermore, our findings point to factors that affect the extent to which task sharing frees up higher-cadre HCWs and reduces human resource costs. Benefits from task sharing depended, first, on how much of a fixed time cost that could include non-activity specific time need (e.g., administration) can be shifted between HCWs; second, on the time added by a workflow interruption; and third, on the salary differences between the HCWs sharing tasks.

### Strengths and limitations

The strengths of this study include the recording of HCW time use and activity in their actual work environment. Furthermore, we captured HCW from different cadres and different facilities over an extended period. The study limitations include, first, that we asked HCW to self-record times before and after providing PrEP activities. We can therefore not exclude that recorded times were estimated. Second, we estimated an average human resource need for PrEP activities in a PrEP demonstration project. We adjusted for few covariates; in particular, characteristics of the process in which these PrEP activities were provided to assess workflow interruptions. The generalizability of our findings depends on whether human resource needs for PrEP activities systematically vary with unassessed factors, like the person, HCW cadre, or health facility providing PrEP. Our supplementary analysis showed, for instance, that the time spent on PrEP provision decreased with the time since the introduction of PrEP. This could reflect learning over time and influence how our findings translate to other care settings and future PrEP provision. On the other hand, not adjusting for experience with PrEP beyond excluding data from the first two months might prevent over-adjustment in our analysis, as clinical practice involves ongoing learning and re-learning due to advancement of care and staff turnover. A third set of limitations regards the data collection. The time-and-motion recording was subject to the availability of a research assistant to visit a clinic and deviated from pre-scheduled study days. In addition, the travel distance between a clinic and the study office in Mbabane affected when research assistants started and ended the time-and-motion recording. Furthermore, the time-and-motion form was attached to the client file, but client files may not have been always available at the right time at the right place to record all PrEP-related interactions. Each of these aspects might have introduced a recording bias to the time-and-motion data and our derived human resource need and cost estimates. Fourth, PrEP guidelines in Eswatini have changed the indications for creatinine testing over time, which likely reduced the HCW time needed for blood testing during PrEP care after the demonstration project.

## Conclusions

A global shortage of skilled HCWs could slow the expansion of PrEP to all people at substantial risk of HIV infection. We estimated the human resources needs and costs required to provide oral PrEP through nurse-led primary care clinics in Eswatini. The estimated HCW time needs for PrEP initiation and follow-up visits were in the lower range of time needs previously reported for the provision of PrEP in other settings. We also assessed opportunities and implications of task shifting and sharing in five scenarios. Sharing the provision of PrEP activities among different HCW cadres showed potential to free up nurse time and reduce HCW costs in PrEP care substantially, but workflow interruptions can increase the overall human resource need. As assessed and unassessed uncertainty around the benefits of task sharing remain, a cautious approach to task sharing in PrEP care could combine the creation of an enabling environment for task sharing with workflow and quality monitoring and evaluation. Finally, to value the full potential benefits of task sharing, the use of the freed up worktime of higher-cadre HCWs should also be considered.

## Supplementary Information

**Additional file 1. **Supplement A: Supplementary tables

**Additional file 2. **Supplement B: Commented Standard Operating Procedure No. 2: 'SIHLOMILE' Expanding HIV Prevention through Pre-Exposure Prophylaxis

**Additional file 3. **Supplement C: Implementation Guide for PrEP demonstration projects in Swaziland

**Additional file 4. **Supplement D: Time-and-motion form used for data collection in a PrEP demonstration project in Eswatini

## Data Availability

The data and code that support the findings of this study are openly available in heiDATA at 10.11588/data/JABBCS.

## Abbreviations

HCW: Health care worker
HIV: Human immunodeficiency virus
HTS: HIV testing services
IQR: Interquartile range
PrEP: HIV pre-exposure prophylaxis
UI: Uncertainty interval
