# Supplementary material for: Human resource needs and costs for HIV pre-exposure prophylaxis provision in nurse-led primary care in Eswatini and opportunities for task sharing﻿
Source: Hum Resour Health. 2022 Oct 23;20:75. doi: 10.1186/s12960-022-00770-9 (PMC9590230; doi:10.1186/s12960-022-00770-9)
Supplement: Supplementary file 1 — Additional file 1. Supplement A: Supplementary tables [file 12960_2022_770_MOESM1_ESM.docx]

**Supplement A:** Supplementary tables

**Table S1:** Overview of previous time-and-motion studies on PrEP provision

| **Study** | **Study setting** | **PrEP group** | **PrEP activity** | **N** | **Mean (range)** | **Median (IQR)** |
| --- | --- | --- | --- | --- | --- | --- |
| Peebles et al. 2021 [1] | Kenya, 25 high-volume HIV-1 care clinics, 8 selected for TM, 02/2018, 03/2019 | Serodiscordant couples | PrEP initiation visits | 14 |  | 43 (38–46) |
|  |  |  | Follow-up visits | 26 |  | 33 (24–35) |
|  |  |  | Refill-only | 6 |  | 15 (7–20) |
| Irungu et al. 2019 [2] | Kenya, public HIV care clinics, 03/2014–05/2014 | Serodiscordant couples | Screening | 1 | 13 |  |
|  |  |  | Enrollment | 2 | 51 |  |
|  |  |  | Follow up | 15 | 12 |  |
| Ying et al. 2015 [3] | Uganda, Kasangati Health Centre, 01/2014–03/2014 | Serodiscordant couples at high risk of HIV infection | Screening (as studied) | 7 | 36 |  |
|  |  |  | Enrolment (as studied) | 5 | 78 |  |
|  |  |  | Follow-up (as studied) | 18 | 36 |  |
|  |  |  | Screening (Ministry of Health scenario) |  | 24 |  |
|  |  |  | Enrolment (Ministry of Health scenario) |  | 36 |  |
|  |  |  | Follow-up (Ministry of Health scenario) |  | 24 |  |
| Hughes et al. 2020 [4] | Zimbabwe, University of Zimbabwe College of Health Sciences Clinical Trials Research Centre (UZCHS-CTRC) Zengeza 3 Clinical Research Site, 03/2017–07/2019 | HIV-discordant couples | HIV prevention counseling | 2 | 8 (6–10) |  |
|  |  |  | PrEP Initiation | 5 | 13.6 (8–20) |  |
|  |  |  | PrEP management/maintenance | 2 | 5.5 (3–8) |  |
|  |  |  | PrEP adherence counseling and monitoring | 2 | 8.5 (7–10) |  |
|  |  |  | On-site HIV test (including pre-testing, testing, and follow-up) | 15 | 8.3 (5–16) |  |
| Roche et al. 2021 [5] | Kenya, 4 public clinics introducing one-stop shop (OSS) service model, 01/2020–11/2020 | Adolescent girls and young women | Provider contact time (PrEP initiation and follow-up visits at OSS) |  |  | 23 |
|  |  |  | Provider contact time (before OSS) |  |  | 24 (10–38) |
|  |  |  | Provider contact time (after OSS) |  |  | 22 (5–40) |
| Roberts et al. 2019 [6] | Kenya, 16 maternal and child health and family planning clinics participating in PrEP Implementation in Young Women and Adolescents (PrIYA) program, 8 selected for TM, 11/2017–06/2018 | Young women and adolescents | Rapid Assessment Screening Tool (RAST; any) | 63 | 7.5 (2–21) | 7 (4–11) |
|  |  |  | Screening and counseling (declined) | 39 | 7.4 (2–21) | 6 (4–10) |
|  |  |  | Screening and counseling (initiated) | 24 | 7.8 (2–18) | 7.5 (4.75–11) |
|  |  |  | Post-RAST PrEP (initiated) | 23 | 12.7 (3–35) | 11 (8–14.5) |
|  |  |  | Total time (initiated), excluding self-test | 23 | 20.6 (8–50) | 19 (12–26) |
|  |  |  | PrEP follow-up | 4 | 9 (7–12) | 8.5 (7.75–9.75) |
|  |  |  | Creatinine | 23 | 3.5 (1–8) | 3 (2–4) |
|  |  |  | HIV testing and counselling | 20 | 13.4 (8–24) | 12 (10–15.25) |
| Mudimu et al. 2022 [7] | South Africa, 2 community-based HIV testing (CBCT) centers, 11/2018–11/2019 | Adolescent girls and young women | PrEP initiation | 9 |  | 51 (43–63) |
|  |  |  | Scheduling calls and sending a text message | 10 |  | 2 (1–3) |
|  |  |  | Standard care refill visit | 15 |  | 17 (15–21) |
|  |  |  | Club visits (monthly except quarterly) | 4 |  | 43 (31–67) |
|  |  |  | Club visits (quarterly) | 4 |  | 57 (47–62) |
|  |  |  | Individual visits (monthly) | 10 |  | 30 (17–34) |
|  |  |  | Individual visits (quarterly) | 8 |  | 46 (36–55) |
| Pintye et al. 2018 [8] | Kenya, 16 facilities participating PrEP Implementation in Young Women and Adolescents (PrIYA) program for integrating PrEP into ANC/PNC visits, 6 selected for TM after offering PrEP in previous 6 months, 2017–2018 | Women in ANC/PNC | Behavioral risk assessment and PrEP counseling (declined) | 10 | 10 (2–15) | 13 (7–15) |
|  |  |  | PrEP activities up to initiation, incl. point-of-care (POC) creatinine testing | 14 | 19 (6–46) | 18 (15–26) |
|  |  |  | POC creatinine testing (willing to initiate) | 16 | 4 (1–8) | 3 (2–4) |
|  |  |  | PrEP-specific activities excluding POC creatinine testing | 14 |  | 15 (14–23) |
| Digre et al. 2021 [9] | USA, TM with up to 8 disease intervention specialists (DIS) of sexually transmitted disease partner services programs newly integrating PrEP referrals to existing services, 02/2018 | Men who have sex with men | PrEP referral and documentation | About 40 h of observed | 40 (12–246) |  |
|  |  |  | Offer PrEP referral during interview |  | 3 (8–15) | 10 (9–12) |
|  |  |  | Document PrEP referral form |  | 4 (1–3) | 3 (2–3) |
|  |  |  | Contact patient |  | 10 (1–15) | 2 (1–5) |
|  |  |  | Contact provider |  | 11 (1–90) | 6 (4–11) |
|  |  |  | Documentation |  | 5 (1–7) | 6 (4–13) |

Based on screening of 13 results from a PubMed search for (“time and motion” OR “time motion”) AND (“pre-exposure prophylaxis” OR “PrEP”) on April 23, 2022. IQR = interquartile range. ANC = antenatal care. PNC = postnatal care. POC = point of care. TM = time-and-motion study.

**Table S2:** Characteristics of the six primary care clinics providing oral PrEP in a demonstration project in the Hhohho region of Eswatini

| **Primary care clinic location** | **Ownership** | **Catch-ment area** | **Clients per month** | **ART clients** | **Nurses** | **HTS counselors** | **Peer supporters** | **Start of PrEP promotion package** |
| --- | --- | --- | --- | --- | --- | --- | --- | --- |
| Ndvwabangeni | Mission | 14868 | 1031 | 101 | 5 | 1 | 4 | 02/2018 |
| Horo | Government | 14317 | 1884 | 761 | 5 | 1 | 4 | 05/2018 |
| Ntfonjeni | Government | 12875 | 1758 | 229 | 4 | 1 | 2 | 02/2018 |
| Ndzingeni | Government | 7445 | 521 | 162 | 5 | 1 | 2 | 05/2018 |
| Hhukwini | Government | 7658 | 905 | 74 | 5 | 0 | 3 | 05/2018 |
| Siphocosini | Mission | 9666 | 1145 | 586 | 5 | 1 | 2 | 02/2018 |

ART = antiretroviral therapy. HTS = HIV testing services. Peer supporters = mother-to-mother mentors or expert clients. Estimated population size in the clinic’s catchment area in 2016. Mean monthly number of primary healthcare visits between August 2016 and July 2017. Total number of clients on antiretroviral therapy between August 2016 and July 2017. A map of the location of the primary care clinics and study office can be viewed under https://goo.gl/maps/qNHKMEpJDvvX9PMz5. *Data source:* Geldsetzer et al. [10].

**Table S3:** Recommended health care worker qualification for PrEP activities in a demonstration project in Eswatini

| **PrEP activity** | **Health care worker cadre** | | |
| --- | --- | --- | --- |
|  | **Nurse** | **HIV testing services counsellor** | **Mother-to-mother mentor or expert client** |
| ***PrEP initiation visit*** | | | |
| Pre-PrEP counseling | Yes | Yes | Yes |
| HIV risk assessment | Yes | Yes | Yes |
| PrEP eligibility screening | Yes | No | No |
| PrEP initiation and first drug supply | Yes^*^ | No | No |
| ***PrEP follow-up visits*** | | | |
| Follow-up counseling and drug refill | Yes^*^ | No | No |

^*^Trained in nurse-led antiretroviral therapy initiation (NARTIS). *Data source:* Eswatini Ministry of Health [11].

**Table S4:** PrEP activities recorded in time-and-motion study by health care worker type

| **Health care worker cadre** | **Pre-PrEP counseling** | **HIV risk assessment** | **PrEP eligibility screening** | **PrEP initiation** | **Follow-up counseling** | **Blood testing** | **All PrEP activities** |
| --- | --- | --- | --- | --- | --- | --- | --- |
| Nursing sister | 3 (2.4) | 3 (2.5) | 4 (5.1) | 4 (6.2) | 4 (4.1) | 0 (0) | 18 (3.0) |
| Nurse | 29 (23) | 27 (22) | 53 (67) | 55 (85) | 51 (53) | 14 (13) | 229 (38) |
| Nurse assistant | 16 (13) | 16 (13) | 5 (6.3) | 4 (6.2) | 5 (5.2) | 8 (7.2) | 54 (9.0) |
| HTS counsellor | 78 (61) | 75 (61) | 17 (22) | 2 (3.1) | 37 (38) | 80 (72) | 289 (48) |
| Phlebotomist | 0 (0) | 0 (0) | 0 (0) | 0 (0) | 0 (0) | 9 (8.1) | 9 (1.5) |
| Mother-to-mother mentor | 1 (0.79) | 1 (0.79) | 0 (0) | 0 (0) | 0 (0) | 0 (0) | 2 (0.33) |
| Total | 127 (100) | 122 (100) | 79 (100) | 65 (100) | 97 (100) | 111 (100) | 601 (100) |

N = 601. ( ) = % of total. No expert client recorded time and motion.

**Table S5:** Estimated and predicted health care worker time for providing PrEP in nurse-led primary care clinics in Eswatini

| **Health care worker time** | **Regression models without covariates** | | **Regression models with covariates** | | | |
| --- | --- | --- | --- | --- | --- | --- |
|  | **Model 1** | **Model 2^†^** | **Model 3^†^** | **Model 4^‡^** | **Model 5** | **Model set 6** |
|  | **Coef. (95% CI)** | **Coef. (95% CI)** | **Coef. (95% CI)** | **Coef. (95% CI)** | **Coef. (95% CI)** | **Coef. (95% CI)** |
| ***PrEP activity (estimated minutes)*** | | | | | | |
| Pre-PrEP counseling | 6.6 (1.1−12.1)^*^ | 3.0 (-1.3−7.2) | 4.3 (-0.14−8.7) | 4.0 (-0.80−8.8) | 3.4 (-0.83−7.6) | 2.6−3.5 (-1.9−7.9) |
| HIV risk assessment | 4.6 (-1.2−10.4) | 1.8 (-2.6−6.1) | 3.3 (-1.4−7.9) | 3.3 (-1.6−8.2) | 3.7 (-0.65−8.1) | 1.7−2.4 (-2.8−6.5) |
| PrEP eligibility screening | 4.6 (0.7−8.4)^*^ | 3.2 (0.0042−6.4)^*^ | 3.8 (0.37−7.2)^*^ | 3.8 (0.46−7.2)^*^ | 3.5 (0.091−6.9)^*^ | 3.0−3.4 (-0.4−6.7)^+^ |
| PrEP initiation | 14 (10−19)^***^ | 8.9 (4.8−13)^***^ | 11 (6.9−16)^***^ | 11 (6.5−16)^***^ | 12 (6.7−16)^***^ | 8.9−9.4 (4.6−13)^+++^ |
| Follow-up counseling | 12 (10−14)^***^ | 4.1 (1.2−7.0)^**^ | 4.7 (1.7−7.7)^**^ | 4.7 (1.7−7.8)^**^ | 5.2 (2.0−8.4)^**^ | 3.9−4.8 (1.0−7.8)^++^ |
| Blood testing | 6.3 (4.5−8.1)^***^ | 3.2 (1.3−5.0)^***^ | 4.0 (1.8−6.2)^***^ | 4.8 (2.5−7.1)^***^ | 4.8 (2.5−7.1)^***^ | 2.9−3.9 (1.1−5.9)^+++^ |
| ***Covariates (estimated minutes)*** | | | | | | |
| Number of PrEP activities provided |  |  | -1.5 (-2.7−-0.30)^*^ | -1.4 (-2.6−-0.19)^*^ | -1.3 (-2.5−-0.050)^*^ | -0.30 (-1.1−0.50) |
| Interrupted workflow |  |  | 3.4 (0.69−6.0)^*^ | 3.3 (0.61−6.0)^*^ | 2.6 (-0.16−5.3) | 1.3 (-0.59−3.1) |
| Month of demonstration project |  |  |  | -0.37 (-0.63−-0.11)^**^ | -0.40 (-0.77−-0.13)^**^ | -0.20 (-0.37−-0.033)^*^ |
| PrEP promotion package |  |  |  | 2.3 (-0.59−5.2) | 2.7 (-0.29−5.7) | -1.0 (-2.8−0.82) |
| Nurse involved in PrEP activity |  |  |  | 0.27 (-2.1−2.6) | -0.077 (-2.5−2.3) | -0.30 (-2.59−1.98) |
| Clinic fixed effects |  |  |  |  | Yes^#^ | 1.8-4.0 (-1.0−6.9) |
| ***Fixed time cost (estimated minutes)*** | | | | | | |
| Constant |  | 8.8 (6.1−12)^***^ | 8.9 (5.8−12)^***^ | 11 (7.4−15)^***^ | 8.4 (4.3−13)^***^ | 5.6−11 (2.0−14)^++^ |
| ***PrEP clinic visit (predicted minutes)*** | | | | | | |
| PrEP initiation visit (minimum) | 36 (34−39)^***^ | 29 (25−32)^***^ | 28 (25−32)^***^ | 24 (19−30)^***^ | 22 (16−27)^***^ | 28 (24−32)^***^ |
| —maximum |  |  | 32 (27−36)^***^ | 37 (31−42)^***^ | 38 (32−44)^***^ | 31 (27−36)^***^ |
| PrEP follow-up visit (minimum) | 18 (16−20)^***^ | 16 (14−18)^***^ | 15 (12−17)^***^ | 11 (7.0−15)^***^ | 8.5 (3.7−13)^***^ | 15 (13−18)^***^ |
| —maximum |  |  | 18 (15−21)^***^ | 23 (19−28)^***^ | 25 (19−30)^***^ | 19 (16−22)^***^ |
| ***Model statistics*** | | | | | | |
| Adjusted R^2^ | 0.83 | 0.24 | 0.26 | 0.27 | 0.28 | 0.27−0.29 |
| AIC/BIC | 1943/1965 | 1894/1920 | 1891/1924 | 1890/1934 | 1892/1954 | 1891−1896/  1920−1939 |

N = 284. ^*^ P<0.05, ^**^ P<0.01, ^***^ P<0.001. AIC = Akaike information criterion. BIC = Bayesian information criterion. ^†^Models selected for main analysis. ^‡^Predicted times per clinic visit for month 1 and 18 of the demonstration project. ^#^Wald test for joint significance P = 0.11. ^+^Highest significance level reached for this independent variable. Model set 6 shows estimates of regression models with each covariate on its own.

**Table S6:** Predicted human resource costs for PrEP initiation follow-up visits with task shifting and sharing

| **Scenario** | **Pre-PrEP counseling** | **HIV risk assessment** | **PrEP eligibility screening** | **PrEP initiation** | **Follow-up counselling** | **Blood testing** | **PrEP initiation visit ($)^#^** | **PrEP follow-up visit ($)^#^** |
| --- | --- | --- | --- | --- | --- | --- | --- | --- |
| Base case | Nurse | Nurse | Nurse | Nurse | Nurse | Nurse | 4.55 [1.52−9.69] | 2.54 [1.07−4.64] |
| Scenario 1 | Nursing sister | Nursing sister | Nursing sister | Nursing sister | Nursing sister | Nursing sister | 7.40 [2.97−13] | 4.14 [2.10−6.41] |
| Scenario 2 | HTS counsellor | HTS counsellor | Nurse | Nurse | Nurse | Phlebotomist | 2.75−3.72 [0.96−7.07] | 1.41−2.24 [0.56−4.01] |
| Scenario 3 | HTS counsellor | HTS counsellor | Nurse | Nurse | Nurse | HTS counsellor | 2.70−3.67 [0.95−6.94] | 1.22−2.19 [0.50−3.89] |
| Scenario 4 | HTS counsellor | HTS counsellor | Nurse | Nurse | Nurse assistant | HTS counsellor | 2.70−3.67 [0.95−6.94] | 1.03−1.61 [0.48−2.43] |
| Scenario 5 | Peer supporter | Peer supporter | Nurse | Nurse | Nurse assistant | HTS counsellor | 2.25−3.52 [0.75−6.05] | 1.03−1.61 [0.48−2.43] |

[ ] = uncertainty interval. HCW = health care worker. HTS = HIV testing services. Peer supporter = mother-to-mother mentor or expert client. ^#^Cost range with fixed time cost based on lowest and highest HCW cadre salary when different HCW cadres provide PrEP activities. **Scenarios 1−3** represent practiced task shifting and task sharing. **Scenarios** **4, 5** represent additional opportunities for task sharing. Time needs estimated based on **Table 2 Model 1**.

**Table S7:** Predicted human resource costs for PrEP activities by health care worker qualification.

| **Human resource costs ($)** | **Nursing sister** | **Nurse** | **Nurse assistant** | **Phlebotomist** | **HTS counsellor** | **Peer supporter** |
| --- | --- | --- | --- | --- | --- | --- |
| ***Unit cost of time*** | | | | | | |
| Salary per minute | 0.26 (0.24−0.27) | 0.16 (0.12−0.20) | 0.11 (0.11−0.12) | 0.063 (0.055−0.072) | 0.047 | 0.014 |
| ***Fixed time cost*** | | | | | | |
| Fixed cost per PrEP visit | 2.28 [1.40−3.27] | 1.40 [0.71−2.37] | 1.00 [0.62−1.42] | 0.56 [0.32−0.86] | 0.42 [0.27−0.57] | 0.13 [0.082−0.17] |
| ***Cost of PrEP activity*** | | | | | | |
| Pre-PrEP counseling | 0.73 [0−2.30] | 0.45 [0−1.67] | 0.32 [0−1.00] |  | 0.13 [0−0.40] | 0.040 [0−0.12] |
| HIV risk assessment | 0.46 [0−2.07] | 0.28 [0−1.50] | 0.20 [0−0.90] |  | 0.085 [0−0.36] | 0.025 [0−0.11] |
| PrEP eligibility screening | 0.60 [0−1.88] | 0.37 [0−1.36] | 0.26 [0−0.82] |  |  |  |
| PrEP initiation | 2.56 [1.04−4.25] | 1.57 [0.53−3.08] | 1.12 [0.46−1.85] |  |  |  |
| Follow-up counselling | 0.82 [0−2.01] | 0.51 [0−1.46] | 0.36 [0−0.87] |  | 0.15 [0−0.35] |  |
| Blood testing | 0.65 [0−1.59] | 0.40 [0−1.15] | 0.28 [0−0.69] | 0.16 [0−0.42] | 0.12 [0−0.28] |  |
| ***Cost of workflow interruption*** | | | | | | |
| Interrupted workflow | 0.87 [0.17−1.64] | 0.53 [0.085−1.19] | 0.38 [0.075−0.71] | 0.21 [0.038−0.43] | 0.16 [0.033−0.29] | 0.048 [0.0098−0.086] |

( ) = range. [ ] = uncertainty interval. Nurse = registered nurse with single or double qualification. HTS = HIV testing services. Peer supporter = mother-to-mother mentor or expert client. Empty cell = health care worker unlikely to provide this PrEP activity. Time needs estimated based on **Table 2 Model 2**.

**References**

1. Peebles, K., et al., *Low costs and opportunities for efficiency: a cost analysis of the first year of programmatic PrEP delivery in Kenya's public sector.* BMC Health Serv Res, 2021. **21**(1): p. 823.

2. Irungu, E.M., et al., *The Incremental Cost of Delivering PrEP as a Bridge to ART for HIV Serodiscordant Couples in Public HIV Care Clinics in Kenya.* AIDS Res Treat, 2019. **2019**: p. 4170615.

3. Ying, R., et al., *Cost-effectiveness of pre-exposure prophylaxis targeted to high-risk serodiscordant couples as a bridge to sustained ART use in Kampala, Uganda.* J Int AIDS Soc, 2015. **18**(4 Suppl 3): p. 20013.

4. Hughes, C.S., et al., *Estimated costs for the delivery of safer conception strategies for HIV-discordant couples in Zimbabwe: a cost analysis.* BMC Health Services Research, 2020. **20**(1): p. 940.

5. Roche, S.D., et al., *A one-stop shop model for improved efficiency of pre-exposure prophylaxis delivery in public clinics in western Kenya: a mixed methods implementation science study.* J Int AIDS Soc, 2021. **24**(12): p. e25845.

6. Roberts, D.A., et al., *The role of costing in the introduction and scale-up of HIV pre-exposure prophylaxis: evidence from integrating PrEP into routine maternal and child health and family planning clinics in western Kenya.* J Int AIDS Soc, 2019. **22 Suppl 4**: p. e25296.

7. Mudimu, E., et al., *Incremental costs of integrated PrEP provision and effective use counselling in community-based platforms for adolescent girls and young women in South Africa: an observational study.* J Int AIDS Soc, 2022. **25**(2): p. e25875.

8. Pintye, J., et al., *Brief Report: Integration of PrEP Services Into Routine Antenatal and Postnatal Care: Experiences From an Implementation Program in Western Kenya.* J Acquir Immune Defic Syndr, 2018. **79**(5): p. 590-595.

9. Digre, P., et al., *Barriers, Facilitators, and Cost of Integrating HIV-Related Activities Into Sexually Transmitted Disease Partner Services in Jackson, Mississippi.* Sex Transm Dis, 2021. **48**(3): p. 145-151.

10. Geldsetzer, P., et al., *A stepped-wedge randomized trial and qualitative survey of HIV pre-exposure prophylaxis uptake in the Eswatini population.* Science Translational Medicine, 2020. **12**(562): p. eaba4487.

11. *Standard Operating Procedure No. 2: 'SIHLOMILE' Expanding HIV Prevention through Pre-Exposure Prophylaxis*. 2017, Mbabane: Eswatini Ministy of Health.
