# Supplementary material for: Human resource needs and costs for HIV pre-exposure prophylaxis provision in nurse-led primary care in Eswatini and opportunities for task sharing﻿
Source: Hum Resour Health. 2022 Oct 23;20:75. doi: 10.1186/s12960-022-00770-9 (PMC9590230; doi:10.1186/s12960-022-00770-9)
Supplement: Supplementary file 2 — Additional file 2. Supplement B: Commented Standard Operating Procedure No. 2: 'SIHLOMILE' Expanding HIV Prevention through Pre-Exposure Prophylaxis [file 12960_2022_770_MOESM2_ESM.pdf]

Supplement B: Commented Standard Operating Procedure No. 2: 'SIHLOMILE' Expanding HIV Prevention through Pre-Exposure Prophylaxis

Ministry of Health Swaziland

'SIHLOMILE' Expanding HIV prevention through Pre-exposure prophylaxis

**STANDARD OPERATING PROCEDURE**	**No. 02**
**PrEP Facility Implementation**	
Effective date: 01/08/2017	Next Review Date: 01/02/2018

	**Name and Position**	**Signature**	**Date**
Author	Allie Hughey<br>Research Associate	[illegible]	19/07/17
Reviewer	Anita Hettema<br>Research Manager	[illegible]	19/07/17
Approver	Sindy Matse<br>Principal Investigator	[illegible]	19/07/017

Revision History

**Ver. No.**	**Revised by (Name & Position)**	**Effective Date**	**Details of Changes**

1. **Purpose**

The purpose of this standard operating procedure (SOP) is to outline the facility procedures related to the 'Sihlomile' Expanding HIV prevention through Pre-exposure prophylaxis ("the study").

2. **Scope**

This SOP will cover the processes and required documentation related to study procedures that are outside the standard of care. This SOP is applicable for procedures involved with:

- Sensitization of potential PrEP clients within the health facility
- HIV risk assessment screening
- PrEP eligibility screening
- PrEP initiation
- PrEP follow-up visits
- Monthly reporting

3. **Staff to whom the SOP is applicable**

All facility staff, including but not limited to:

- Medical officers
- Nurses
- Counsellors and expert clients
- Mothers to mothers (M2M)
- Phlebotomists
- Nurse Mentors

4. **Materials required**

- PrEP flyers, posters and palm cards (when available)
- PrEP client flow chart
- PrEP register
- PrEP client files
- PrEP client appointment cards
- HIV risk assessment & eligibility (RA&E) forms
- PrEP monthly reporting form

5. **Study procedures at demonstration sites**

PrEP activities included in costing

5.1. Sensitization of potential PrEP clients within the health facility

5.1.1. Routine morning education sessions in the waiting area of the study sites will be used to communicate general messages about PrEP and its availability to potential clients. (See Annex 2 for a sample script for sensitization of potential PrEP clients).

5.1.2. At each service delivery point in the facility, potential clients will be educated and counselled by health care workers about the use, risk and benefits of PrEP.

5.1.3. PrEP education and counselling can be done by HIV Testing Service (HTS) counsellors, expert clients, Mothers 2 Mothers (M2M), nurses and medical officers (MOs).

Pre-PrEP counseling

5.1.4. See Annex 2 for key counselling messages for PrEP.

5.2. HIV risk assessment screening

5.2.1. HIV risk assessment screening may be performed by an expert client, HTS counselor, M2M, nurse or MO (collectively referred to as HCWs below).

HIV risk assessment

5.2.2. HCWs should make every effort to establish rapport with potential PrEP clients, provide adequate privacy and offer assurances of confidentiality.

5.2.3. After a client has been sensitized about PrEP and expresses interest in knowing more, they will be offered an HIV risk assessment screening. The purpose of the risk discussion will be explained to the client.

5.2.4. The HCW will use a new HIV Risk Assessment & PrEP Eligibility Screening (RA&E) form for each new client.

5.2.5. Verbal consent will be obtained and documented on the RA&E form by the HCW prior to beginning the screening.

5.2.6. The HCW will document the client demographics on the RA&E form and qualified HTS providers will proceed with an HIV test for the client.

5.2.6.1. If the HCW who initiated the HIV risk assessment is not a qualified HTS provider, he/she will refer the client to HTS to conduct the HIV test and continue with the HIV risk assessment.

5.2.6.2. Clients with a reactive HIV test will immediately be linked to ART.

5.2.7. For clients that are confirmed to be HIV negative, the HCW will continue with the HIV risk assessment and determine the client's HIV infection risk.

5.2.7.1. If a client is determined to be *at substantial risk for HIV infection and interested in PrEP*, the HCW will proceed to PrEP eligibility screening.

5.2.7.2. If a client is determined to be *at substantial risk for HIV infection but NOT interested in PrEP* or the client is *not at substantial risk for HIV infection*, the HCW will counsel the client on other HIV prevention services.

5.2.8. All clients who consent to receive an HIV risk assessment will be asked to consent to a set of 5 additional questions for the study. The HCW will read the consent statement for the additional questions to the client and document the client's verbal consent before asking the additional questions.

5.2.9.If the client will not proceed to eligibility screening, the completed RA&E form and additional questions will be filed in the appropriate secured location.

HIV risk assessment

5.3. PrEP eligibility screening

5.3.1. PrEP eligibility screening must be performed by a nurse or MO.

5.3.2. Eligibility screening will include:

- Symptoms of acute HIV infection
- Symptoms of STI
- HBsAg test and Hepatitis B vaccination status
- Serum creatinine test
- Check for non-communicable diseases

5.3.3.A blood sample for serum creatinine testing MUST be taken prior to PrEP initiation.

5.3.3.1. If sample transport is not occurring on that day but will collect samples the following day, the blood sample can be taken and *stored in the refrigerator (2-4°C) after clotting* and sent with sample transport the next day.

5.3.4.Unavailability of creatinine clearance results is not a contraindication for PrEP in most clients. For clients in the following situations, it is recommended to defer PrEP initiation until creatinine results are available:

- Diabetes Mellitus
- Hypertension → take baseline BP
- BMI < 18.5
- Age above 50 years
- Suspected renal disease
- Clients who are dehydrated

5.3.5. After the nurse or MO completes eligibility screening procedures, he/she will complete the eligibility checklist on the RA&E form to conclude if the client is eligible

PrEP eligibility screening and blood testing

for PrEP initiation. A client must have a 'yes' response for all 9 eligibility questions to be eligible for PrEP initiation.

5.3.6. If client is eligible, the nurse or MO will proceed to the consent process for PrEP initiation. (section 5.4)

5.3.7. If client is not eligible for PrEP, the nurse or MO will offer other HIV prevention services and complete the RA&E form before filing it in the appropriate location.

PrEP eligibility screening

5.4. Written consent for PrEP initiation

5.4.1. ALL clients who wish to initiate PrEP MUST provide written informed consent before receiving PrEP drugs.

5.4.2. The nurse or MO will provide the client with the participant information sheet for clients opting in to start PrEP. They will also answer any questions the client may have or clarify anything that is unclear.

5.4.3. The client will then complete and sign the participant informed consent for PrEP initiation form.

5.4.4. The nurse or MO must sign the informed consent form as a witness and give the duplicate copy of the signed consent form to the client before proceeding to PrEP initiation.

5.4.5. If the client consents to PrEP initiation, the nurse or MO will indicate 'yes' in the 'informed consent' portion of the RA&E form before proceeding to PrEP initiation.

5.4.5.1. If a nurse conducting the eligibility screening and consent process is not a NARTIS-trained, he/she will refer the client to the appropriate service provider for PrEP initiation.

5.4.6. If the client does not consent to PrEP initiation, the nurse or MO will offer other HIV prevention services and ensure complete documentation of the RA&E form, including the 'informed consent' portion. The nurse or MO will then file the RA&E form in the appropriate location.

5.5. PrEP initiation

PrEP initiation (PrEP uptake)

5.5.1. PrEP initiation needs to be completed by a NARTIS-trained nurse or MO.

5.5.2. Prior to initiation, the NARTIS-trained nurse or MO needs to ensure the client is willing and committed to start PrEP and that documentation related to HIV risk assessment, eligibility screening and informed consent has been completed.. Additionally, no client can be initiated on PrEP unless HIV testing done on the same day confirms an HIV negative result.

5.5.3. The NARTIS-trained nurse or MO will document the PrEP client in the register and generate a PrEP ID.

5.5.3.1. PrEP ID consist of 3 letter facility code, Client sequential number and Month/ Year that client is initiated on PrEP.

5.5.4. A PrEP client file should now be opened.

5.5.4.1. The NARTIS-trained nurse or MO will complete the information on the front and inside cover of the PrEP file, including the PrEP ID and client demographic information.

5.5.4.2. The NARTIS-trained nurse or MO will secure the original signed consent form and the completed RA&E form inside the PrEP file.

5.5.4.3. The NARTIS-trained nurse or MO will complete the first row of the PrEP follow-up visit form with initiation visit details and any additional comments on the notes sheet, if necessary.

5.5.5. Clients will be provided with 28 days of TDF/3TC and assigned a follow-up appointment 4 weeks in the future.

5.5.6. The NARTIS-trained nurse or MO will complete the PrEP client appointment card with the client's PrEP ID, facility and date of PrEP initiation as well as the next visit date and provide to the client.

5.5.7. At the end of each day, the follow-up visits for new PrEP clients will be appointed in the facility appointment register.

PrEP initiation

5.6. PrEP follow-up visits

5.6.1. One month follow-up visit

5.6.1.1. The NARTIS-trained nurse or MO will retrieve the correct PrEP client file and ask for the client's PrEP appointment card.

5.6.1.2. All details about the client's one month follow-up visit must be recorded by the NARTIS-trained nurse or MO on the follow-up form in the PrEP client file.

5.6.1.3. The following visit procedures must occur at the one month follow-up visit:

- HIV testing and counselling
- Risk reduction counselling
- Adherence assessment and counselling
- Identify possible barriers to good adherence
- Assess tolerability, side effects and effective use
- Actively manage side effects
- Assess need for contraceptives
- Review of baseline laboratory results
- Offer HBV vaccination if available and HBsAg negative
- STI screening (clinical exam and rapid test/ RPR when indicated)
- HIV risk review and assessment of PrEP continuation
- Calculate creatinine clearance from baseline serum creatinine result

5.6.1.4. If baseline Hepatitis B screening and creatinine results were not available at initiation, laboratory results should be reviewed and recorded in the PrEP follow-up visit form at this time.

5.6.1.5. If there are no side effects, adherence barriers or abnormal laboratory results identified, the NARTIS-trained nurse or MO will give the client a 2 month refill for PrEP.

5.6.1.6. The NARTIS-trained nurse or MO will document the next visit date in the client's appointment card.

5.6.1.7. The NARTIS-trained nurse or MO will store the PrEP client file in the appropriate file location.

PrEP follow-up and blood testing
– 1-month visit
– 3-monthly visit
– 6-monthly visit

5.6.2. Follow-up maintenance visits

5.6.2.1. The NARTIS-trained nurse or MO will retrieve the correct PrEP client file and ask for the client's PrEP appointment card.

5.6.2.2. Follow-up maintenance procedures by The NARTIS-trained nurse or MO will include the following interventions based on the number of months the client has been on PrEP:

Intervention	Months on PrEP						
	1	3	6	9	12	15	18
HIV testing and counselling	X	X	X	X	X	X	X
Risk reduction counselling	X	X	X	X	X	X	X
Adherence assessment and counselling →Identify barriers to good adherence	X	X	X	X	X	X	X

Assess tolerability, side effects and effective use	X	X	X	X	X	X	X
Actively manage side-effects	X	X	X	X	X	X	X
Assess need for contraceptives	X	X	X	X	X	X	X
Review of baseline laboratory results (if not done previously)	X						
Offer HBV vaccination if available and HBsAg negative	X						
STI screening (clinical and use rapid test/ RPR when indicated)	X	X	X	X	X	X	X
HIV risk review and assessment of PrEP continuation			X		X		X
Serum creatinine →Calculate creatinine clearance			X		X		X

PrEP follow-up and blood testing

5.6.2.3. All details about the follow-up visits must be recorded by the NARTIS-trained nurse or MO in the PrEP client follow-up form in the client file.

5.6.2.4. The NARTIS-trained nurse or MO will schedule follow-up maintenance visits every 3 months after the initial 3 months on PrEP and document the follow-up visit date in the client's appointment card.
