# Supplementary material for: Human resource needs and costs for HIV pre-exposure prophylaxis provision in nurse-led primary care in Eswatini and opportunities for task sharing﻿
Source: Hum Resour Health. 2022 Oct 23;20:75. doi: 10.1186/s12960-022-00770-9 (PMC9590230; doi:10.1186/s12960-022-00770-9)
Supplement: Supplementary file 3 — Additional file 3. Supplement C: Implementation Guide for PrEP demonstration projects in Swaziland [file 12960_2022_770_MOESM3_ESM.pdf]

SIYINQABA

IMPLEMENTATION GUIDE FOR PrEP DEMONSTRATION PROJECTS IN SWAZILAND

IMPLEMENTATION GUIDE FOR PrEP DEMONSTRATION PROJECTS IN SWAZILAND

July 2017

ACKNOWLEDGMENTS

This implementation guide has been developed through the collaborative effort of many individuals and organizations. The process, which included a review of documents from the World Health Organization and other countries, was led and coordinated by the Ministry of Health through Swaziland National AIDS Program (SNAP).

The Ministry of Health is thankful to the members of thematic technical working groups—Key Populations, Prevention, and Care and Treatment—who worked tirelessly to provide invaluable strategic information for shaping the framework to guide the implementation of the demonstration projects. The value of the oversight function of the task team cannot be overstated as members provided key technical inputs that moved the process forward.

The ministry is indebted to all development bilateral and multilateral partners, implementing partners, civil society organizations (CSOs), and local communities who collectively contributed to the adaptation of this document, and extends sincere gratitude to Robin Eakle for technical guidance.

FOREWORD

In recent years, evidence has shown that early initiation of antiretroviral therapy (ART) is associated with better individual patient level outcomes including reduced risk of death and severe HIV-associated illness, and broader population level prevention benefits.

Furthermore, studies have demonstrated the efficacy of pre-exposure prophylaxis (PrEP) among various populations, setting the agenda for expanded biomedical tools for HIV prevention. This evidence and wider availability of safer efficacious medicines, including increasing access to monitoring tools and technologies, provide opportunities to expand the use of antiretroviral drugs (ARVs) for prevention. The recommendations presented in this implementation guide are largely in line with the World Health Organization *Consolidated Guidelines on the Use of Antiretroviral Drugs for Treating and Preventing HIV Infection: Recommendations for a Public Health Approach*.

This guidance document will provide the much-needed motivation and framework to conduct the PrEP demonstration projects in Swaziland.

Dr. S. V. Magagula
Director of Health Services

Contents

Introduction to PrEP 1
What is PrEP 1
PrEP in Swaziland 1
PrEP regimens 1

Getting started on PrEP **2**
PrEP sensitization and education 2
Priority Groups for PrEP 3
Who Should Provide PrEP and Where 4
Indications for PrEP 4
Risk Behavior Assessment 5
Eligibility for PrEP 6
Contraindications for PrEP 7
PrEP Initiation and Follow-Up Visits 7
Side Effects 12

Management of clients on PrEP **13**
Clients with a recent HIV exposure and currently on PEP 13
Clients previously on PrEP coming for re-initiation 13
Clients with a hepatitis B co-infection 13
Management of creatinine elevation 13
Management of HIV seroconversion 14
Management of adverse drug reactions (ADR) 14
Stopping PrEP 14
Management of clients requesting a transfer out 15
Management of clients transferring in 15

Key counselling messages **16**
Documentation and data management **19**
PrEP Data Collection Tools **19**
Flowchart for PrEP Implementations **21**

ACRONYMS

**3TC**	Lamivudine
**ADR**	Adverse drug reaction
**ART**	Antiretroviral therapy
**BMI**	Body mass index
**BP**	Blood pressure
**CMS**	Central medical stores
**FDC**	Fixed-dose combination
**FTC**	Emtricitabine
**HBsAg**	Hepatitis B surface antigen
**HBV**	Hepatitis B virus
**HCW**	Health care worker
**HIV**	Human immunodeficiency virus
**HTS**	HIV testing service
**LFT**	Liver function test
**LMP**	Last menstrual period
**M2M**	Mothers 2 Mothers
**MO**	Medical officer
**NARTIS**	Nurse-led ART Initiation in Swaziland
**PEP**	Post-exposure prophylaxis
**PO**	Per oral
**PrEP**	Pre-exposure prophylaxis
**RPR**	Rapid plasma regain
**STI**	Sexually transmitted infection
**TB**	Tuberculosis
**TDF**	Tenofovir disoproxil fumarate
**TFO**	Transfer out
**WHO**	World Health Organization

Introduction to PrEP

What is PrEP

Pre-exposure prophylaxis (PrEP) is a new, efficacious HIV prevention intervention. It involves the use of antiretroviral drugs (ARVs) by people who are not infected with HIV to prevent acquisition of HIV. World Health Organization (WHO) has recommended that PrEP should be offered to people at substantial risk of HIV infection as part of a combined HIV prevention approach. PrEP should not displace or undermine the use of other effective and well-established HIV combination prevention interventions. PrEP should be promoted as an additional prevention choice among people at risk in conjunction with other appropriate prevention methods.

PrEP in Swaziland

In Swaziland, oral PrEP will be implemented in 2017 through demonstration projects. Three demonstration projects will be used to assess the operationalization of PrEP as an additional HIV prevention method offered in public and private facilities. This document provides clinical guidance for the implementation of PrEP in Swaziland in the absence of a national document and is based on WHO recommendations included in the *Consolidated Guidelines on the Use of Antiretroviral Drugs for Treating and Preventing HIV Infection* (second edition, 2016). Only facilities that are participating in one of the demonstration projects will be allowed to prescribe PrEP to HIV-negative individuals identified as at risk for acquiring HIV, based on specific study protocols and standard operating procedures.

PrEP does not eliminate the risk of HIV infection. It does not prevent STIs or unintended pregnancies. It should, therefore, be offered as part of a combination prevention package that includes risk reduction counselling, HIV testing, condoms and lubricants, STI screening and treatment, and contraception.

PrEP regimens

The recommended ARV regimen for use as PrEP in Swaziland:

- tenofovir (TDF) 300 mg and emtricitabine (FTC) 200 mg, orally, given as a fixed-dose combination (FDC)
- tenofovir (TDF) 300 mg and lamivudine (3TC) 300 mg, orally

Getting started on PrEP

PrEP SENSITIZATION AND EDUCATION

Guiding Principles

The implementation of PrEP as an additional HIV prevention approach in Swaziland will be governed by the following fundamental principles in line with public health and a human rights-based approach to delivering universal health services.

Accessibility

PrEP implementation must ensure that PrEP is accessible to those who need it. This includes people who currently have limited access to HIV prevention services, are at increased risk of HIV infection, and/or are vulnerable in accessing and using other HIV prevention methods.

Affordability

Given that a majority of the target population is classified as vulnerable, affordability of services is crucial. It will therefore be important that PrEP services, including the provision of PrEP drugs, are provided free of charge.

Participation and involvement

The service delivery modalities for PrEP should therefore ensure the involvement and participation of the beneficiaries.

Acceptability

Provision of PrEP must be sensitive to the culture and way of life of the target populations and communities. This includes minimization of any approaches that subject people to stigma and discrimination.

Safety and quality of PrEP service delivery

Taking PrEP services to the people in need requires the maintenance of strict quality assurance and compliance with the clinical monitoring guidance.

Informed Consent
People accessing PrEP must give informed consent. Therefore, PrEP services should be offered to clients following provision of adequate and relevant information and counselling.

Confidentiality
Provision of PrEP must be confidential and this includes confidentiality of health records by providers. However, shared confidentiality with a partner, family members, and/or peers should be promoted.

Linkages to other services
Clients enrolled in PrEP services should be linked to other services that they may require and appropriate systems should be developed to track and monitor them.

PRIORITY GROUPS FOR PrEP

The following target groups are considered for the PrEP demonstration project:

- *Young women (16 to 25 years):* HIV incidence is at approximately 4 percent for 18- to 24-year-old females, which is three to four times higher than males in the same age group (Swaziland HIV Incidence Measurement Survey [SHIMS, 2010]). This age group also has a high rate of gender-based violence; 38 percent having experienced sexual violence during their lifetime. In addition, many women transition through well-defined periods of increased risk of HIV infection when condom use is not possible (e.g., when they want to conceive) or reject condoms for whatever reason (e.g., in adolescence).

- *Pregnant and lactating women*: In HIV-negative women, pregnancy is a period of elevated risk of HIV infection, and infection acquired during pregnancy may be more likely to be transmitted to the fetus than a pre-existing infection.

- *Serodiscordant couples*: PrEP can protect the uninfected partner in a serodiscordant relationship with an HIV-infected partner who may not be enrolled in antiretroviral therapy (ART) or is on ART but still has a high viral load.

- *Sex workers*: Female sex workers (FSWs) are at heightened risk of HIV infection secondary to biological, behavioral, and structural risk factors.
- *Men who have sex with men*: Men who have sex with men (MSM) have a 19-fold higher burden of HIV infection. MSM are at increased risk of HIV due to the higher probability of HIV transmission during anal intercourse.
- *Clients with sexually transmitted infections*
  Sexually transmitted infections (STIs) are indicators of risk of sexual acquisition of HIV. A new diagnosis of genital ulcers (syphilis or genital herpes) is a strong predictor of HIV risk among couples/partners.

WHO SHOULD PROVIDE PREP AND WHERE

PrEP in Swaziland will be prescribed by health care professionals who have completed training on the national guidance for the use of ARVs for PrEP with support from lay cadres who should provide counselling to support PrEP provision. PrEP implementation will be integrated into facilities that are already providing sexual and reproductive health (SRH) and HIV services and outreach services. The lay cadres (e.g., expert clients, HTS counsellors, peer outreach workers, community health workers), specifically in primary care clinics and mobile and fixed-site community settings, will be trained to create demand and to support adherence counselling. Training will be through existing mentoring and supervision activities.

INDICATIONS FOR PrEP

PrEP is offered to sexually active HIV-negative individuals who are at significant risk of acquiring HIV infection. Clients must meet the following criteria before initiating PrEP:

- Sexual partner is known to be HIV positive and not on ART, or on ART less than six months, or suspected poor adherence to ART, or most recent viral load is detectable
- Sexual partner/s are of unknown HIV status and are at high risk for HIV infection (has multiple sexual partners, has had STIs, engages in transactional sex, injects drugs, from high HIV burden settings)
- History of recent sexually transmitted infection

- Engaging in transactional sex
- Recurrent use of post-exposure prophylaxis
- History of habitually having sex while under the influence of alcohol or recreational drugs
- Inconsistent or no condom use, or unable to negotiate condom use during intercourse with persons of unknown HIV status
- Injection drug use where needles and syringes are shared
- Serodiscordant couples trying to conceive

RISK BEHAVIOR ASSESSMENT

Providers should make every effort to establish rapport with potential PrEP clients, provide adequate privacy, and offer assurances of confidentiality. PrEP should be offered after assessment to establish eligibility, readiness for effective use, commitment to adhere to required follow-up, and absence of contraindications to TDF and/or FTC.

Explain the purpose of the risk discussion, and be sensitive to the fact that it is a personal, private, and intimate discussion. A risk assessment should not become a barrier for PrEP.

Risk assessment questions include:

In the past SIX months:

- Have you had unprotected (condom-less) sex?
- Have you had sex with partners who are HIV positive or whose HIV status you did not know?
- Have you had a sexually transmitted infection?
- Have you been using post-exposure prophylaxis (PEP)?
- Have you had sex under the influence of alcohol and/or drugs?
- Have you experienced or do you expect any situations which you consider to be risky for acquiring HIV?

Any "yes" answer to the above questions from a person in a high incidence setting should prompt a discussion on the individual risks and benefits of PrEP.

RISK REDUCTION COUNSELLING

Risk reduction counselling is a behavioral intervention that attempts to decrease an individual's chances of acquiring HIV and other STIs, and should be implemented with counselling in HIV prevention, sexual and reproductive health, and contraception at all follow-up visits for PrEP users.

The main objective of risk reduction counselling is for clients to assess individual risk and set realistic goals for behavior change that could reduce their risk of contracting HIV and other STIs, as well as prevent unwanted pregnancies. This counselling, which is most effective when nonprejudicial and user-centred, can be provided by any trained health care provider and should address the following points:

- Explore the context of the client's specific sexual practices and psychosocial status, and assist client in recognizing any of their behaviors that are associated with higher risks for HIV infection. Health care providers should also be aware that clients might not always perceive their own risk or may be in denial about it.
- Identify the sexual health protection needs of the potential PrEP user and reflect on what their main concerns appear to be.
- Strategize with the client on how they can manage these concerns or needs.
- Agree on which strategies the client is willing to explore and provide guidance on how to implement them.

ELIGIBILITY FOR PrEP

Providers should educate and counsel potential PrEP users about PrEP, which should always be provided as part of a combination prevention package.

ELIGIBILITY CRITERIA FOR PrEP

- Confirmed HIV negative (rapid antibody testing following the HTS algorithm on the day of PrEP initiation is adequate confirmation of HIV-negative status)
- Does not have a current or recent (within past month) illness consistent with acute HIV infection (fever, sore throat, muscle or joint pains, swollen glands, diarrhea, or headache) in combination with a preceding high-risk exposure for HIV

- Assess readiness to adhere to PrEP and willingness to attend follow-up evaluations including repeat HIV testing and monitoring for side effects
- No contraindication to use of TDF +/- FTC/3TC

CONTRAINDICATIONS FOR PrEP

PrEP should not be provided to people with:

- Signs of acute HIV seroconversion (flu-like symptoms, sore throat, lymphadenopathy, fever, skin rash)
  - Defer PrEP for four weeks and retest
  - Exclude TB
- Creatinine clearance less than 60 ml/min
- Contraindications for TDF as per national guidelines
- Bodyweight less than 40 kg
- Age less than 16
- Unwilling or unable to adhere to daily PrEP or to come to scheduled follow-up visits
- Unable to provide written consent for PrEP

PrEP INITIATION AND FOLLOW-UP VISIT

Initiation visit

- Prior to initiating PrEP, the health care worker (HCW) needs to ensure the client is willing and committed to start PrEP, and documentation related to risk assessment and eligibility screening has been completed.
- Although PrEP initiation needs to be done by NARTIS-trained nurses, the education, counselling, and preparation for clients to start PrEP can be done by expert clients, HTS counsellors, and/or Mothers 2 Mothers (M2Ms).
- No client can be initiated on PrEP unless HIV testing done on the same day confirms an HIV-negative result.

The following table summarizes the investigations and procedures that need to be completed prior to PrEP initiation, including the baseline laboratory investigations.

Category	Investigation/Procedure	Provider
HIV test	▪ To assess HIV infection status	Expert client<br>HTS counsellor<br>M2M<br>Nurse<br>Medical officer
Counselling	▪ Educate about the risk, benefits, and limitations of PrEP<br>▪ Behavior risk assessment<br>▪ Discuss combination prevention and risk reduction<br>▪ Evaluate eligibility, willingness, and readiness to take PrEP<br>▪ Family planning and safer conception counselling, if applicable	
Clinical/ laboratory screening	▪ STI screening (rapid plasma regain [RPR] or syphilis rapid test when indicated)<br>▪ Last menstrual period (LMP); do pregnancy test if indicated<br>▪ Hepatitis B screening<br>→ HBsAg negative: Offer Hepatitis B vaccination if not previously completed at 0, 1 and 6 months<br>→ HBsAg positive: See management of clients with hepatitis B co-infection (page 13)<br>▪ Creatinine test (calculate creatinine clearance)	Nurse<br>Medical officer
PrEP initiation	▪ Assess exclusion criteria for PrEP/contraindications<br>▪ If no contraindications, provide PrEP for 28 days	

For most clients, PrEP can be initiated the same day without waiting for creatinine results. However, in the following situations it is recommended to defer PrEP initiation:

Client Description	Next Steps
Clients with a recent high-risk HIV exposure who could be in the window period	1. Encourage consistent condom use<br>2. If exposure within 72 hours, discuss option of PEP<br>3. Repeat HIV test after window period<br>4. If HIV-negative, initiate PrEP
Clients at risk for renal impairment:<br>• Diabetes mellitus<br>• Hypertension → take baseline BP<br>• BMI less than 18.5<br>• Age above 50 years<br>• Suspected renal disease<br>• Clients who are dehydrated	1. Wait for creatinine result<br>2. Calculate creatinine clearance<br>3. If creatinine clearance > 60 ml/min, initiate PrEP<br>4. If creatinine clearance < 60 ml/min→ see management of creatinine elevation (page 13)

Documentation of PrEP initiation should be done on:

- PrEP register (use generated PrEP ID on PrEP client card and PrEP file)
- Client PrEP appointment card
- Individual client PrEP file

One month follow-up visit

- The first follow-up appointment will be after 28 days, unless the clients presents earlier with side effects.
- This appointment is important to assess PrEP usage, adherence, and potential barriers to effective PrEP use.
- If baseline hepatitis B screening and creatinine results were not available upon initiation, laboratory results should be reviewed during this visit.
  - HBsAg negative: Offer hepatitis B vaccination if not previously completed

- HBsAg positive: See management of clients with hepatitis B co-infection (page 13)
- Creatinine clearance > 60 ml/min: client can continue PrEP, repeat creatinine when client has been on PrEP for 6 months
- Creatinine clearance < 60 ml/min: See management of clients with creatinine elevation (page 13)

- If no side effects, adherence barriers, or abnormal laboratory results identified, the client can be given a two-month refill for PrEP
- If there are side effects, see management of adverse drug reactions (page 14)

Summary of one month follow-up visit procedures
❑ HIV testing and counselling
❑ Risk reduction counselling
❑ Adherence assessment and counselling →Identify possible barriers to good adherence
❑ Assess tolerability, side effects, and effective use
❑ Actively manage side effects
❑ Assess need for contraceptives
❑ Review of baseline laboratory results (if not done previously)
❑ Offer HBV vaccination if available and HBsAg negative
❑ STI screening (clinical and use rapid test/RPR when indicated)
❑ HIV risk review and assessment of PrEP continuation
❑ Serum creatinine → Calculate creatinine clearance

Follow-up maintenance visits

- Maintenance visits will be scheduled every three months after the first three months on PrEP.
- Clients can only continue PrEP upon confirmation of an HIV-negative test.

The follow-up procedures are summarized in the following table:

Intervention	Months on PrEP						
	1	3	6	9	12	15	18
HIV testing and counselling	X	X	X	X	X	X	X
Risk reduction counselling	X	X	X	X	X	X	X
Adherence assessment and counselling →Identify barriers to good adherence	X	X	X	X	X	X	X
Assess tolerability, side effects, and effective use	X	X	X	X	X	X	X
Actively manage side effects	X	X	X	X	X	X	X
Assess need for contraceptives	X	X	X	X	X	X	X
Review of baseline laboratory results (if not done previously)	X						
Offer HBV vaccination if available and HBsAg negative	X						
STI screening (clinical and use rapid test/RPR when indicated)	X	X	X	X	X	X	X
HIV risk review and assessment of PrEP continuation			X		X		X
Serum creatinine →Calculate creatinine clearance			X		X		X

SIDE EFFECTS

The major toxicities associated with TDF/FTC are rare in PrEP exposure to date. Minor side effects are relatively common but are mild and self-limiting if they do occur (approximately one in 10 individuals in the first one to two months), and do not require discontinuation of PrEP.

Minor side effects: gastrointestinal symptoms (diarrhea, nausea, vomiting, and flatulence), which are self-limiting and typically end within first month of use; unintentional weight loss.

Major side effects: renal toxicity and metabolic complications (decreased bone mineral density, which is reversible in adults upon stopping PrEP), extremely small risk of lactic acidosis and hepatic steatosis or steatohepatitis.

Management of clients on PrEP

CLIENTS WITH A RECENT HIV EXPOSURE AND CURRENTLY ON PEP

- Discuss benefits and limitations of PrEP for clients with a recent HIV exposure currently on PEP.
- Link to PrEP services if client is interested and has completed 28 days of PEP and is confirmed HIV-negative.

CLIENTS PREVIOUSLY ON PrEP COMING FOR RE-INITIATION

- Clients can stop PrEP during periods of low risk. Once a client wants to go back on PrEP, repeat all procedures as PrEP initiation visit, excluding the hepatitis B screening if done previously: See PrEP initiation and follow-up visits (page 7).
- Continue with same PrEP file and PrEP ID.
- Document "re-start" in the PrEP file and register.

CLIENTS WITH A HEPATITIS B CO-INFECTION

- PrEP is not contraindicated in clients with HBV infection; if the HBsAg results come back positive, the client can continue with PrEP.
- Clients with HBV infection will need monitoring with additional liver function tests (LFTs).
- Be cautious when stopping PrEP as there is a risk of viral rebound → Check LFTs when stopping PrEP.

MANAGEMENT OF CREATININE ELEVATION

- Repeat an elevated serum creatinine (creatinine clearance < 60 ml/min) with a repeat sample collected on a different day. (Approximately 80 percent of creatinine elevations are self-limiting (without stopping PrEP) and are caused by dehydration, exercise, or diet, or may be a false-positive test result.
- If repeat sample gives creatinine clearance < 60 ml/min:
  - Stop PrEP.
  - Rule out other causes of elevated creatinine (diabetes mellitus, hypertension, liver failure, hepatitis C virus).
  - Repeat creatinine test after one to three months.

- Re-start PrEP (if indicated) once creatinine clearance > 60 ml/min.
- If creatinine clearance does not return to normal levels within three months of stopping PrEP, refer to medical officer (MO).

MANAGEMENT OF HIV SEROCONVERSION

- HIV seroconversion may occur shortly after receiving PrEP although most documented cases were due to pre-existing HIV infection.
- If a client seroconverts while on PrEP:
  - Offer ART as soon as possible after a positive HIV result (if possible without a gap between PrEP and ART)
  - If provider is not comfortable initiating ART, consult MO at mother facility.

MANAGEMENT OF ADVERSE DRUG REACTIONS (ADR)

- All adverse drug reactions should be recorded in client PrEP file and ADR form
- Complete national ADR form
  - one copy to be placed in PrEP file
  - one copy to be sent to pharmacy at other facility
  - one copy to be sent to pharmacovigilance unit of CMS with monthly drug order
- If Grade 1/2 ADR, treat symptomatically
- If Grade 3/4 ADR, consult MO at mother facility
- If PrEP will be discontinued, record outcome in PrEP file and PrEP register

STOPPING PrEP

- PrEP can be stopped for the following reasons:
  - Positive HIV test
  - Request of user
  - Safety concerns
  - No longer at risk
- Ensure adequate documentation of the reason for stopping PrEP.
- If PrEP is discontinued at request of the user, do not be judgmental. Remember, PrEP is a personal decision.
  - Explore risks and alternative prevention/risk reduction strategies
  - Advise client that an HIV test is required to re-start PrEP

  - PrEP should be continued to be used for 28 days after the last exposure
- Ongoing linkage to appropriate HIV prevention services and contraceptive services should be encouraged, as well as the use of other HIV prevention strategies, as needed.

> The duration of PrEP use may vary, and individuals are likely to start and stop PrEP depending on their risk assessment at different periods in their lives – including changes in relationship status, behaviors, and ability to adhere to a PrEP maintenance program. Clients should be advised that an HIV test at minimum should be done before PrEP is recommenced. Clinicians may want to discuss the options of when to discontinue PrEP with their clients.

MANAGEMENT OF CLIENTS REQUESTING A TRANSFER OUT

- PrEP is only offered in selected facilities. If a client wants to move and is motivated to stay on PrEP, inform clients of other PrEP participating pilot sites they may transfer to.
- Complete national referral tool and include PrEP start date, regimen, and laboratory investigations.
- Record outcome in PrEP register and client file: TFO.
- Put one copy of referral tool in PrEP file of client, send one copy to receiving facility, give one copy to client to take to receiving facility.

MANAGEMENT OF CLIENTS TRANSFERRING IN

- Place transfer letter/referral letter in client file.
- Indicate original PrEP start date in client file.
- Enter client details in original cohort in PrEP register.
- Write transfer-in date and original facility in comment section.
- Repeat PrEP procedures as for new client, including HIV testing: See PrEP initiation and follow-up visits (page 7).

Key counselling messages

Ongoing education and counselling for clients considering PrEP or clients already on PrEP is important to ensure effective use and should include the following:

Effectiveness	PrEP is highly effective if you take it as prescribed. The overall reduction in HIV risk has been more than 90 percent in clinical trials when PrEP was used consistently.
Ways to support adherence	Taking PrEP each day is easiest if you make taking the tablets a daily habit, linked to something you do every day without fail. For example, you could take PrEP when you brush your teeth (either in the morning or evening).<br>If you forget to take a tablet, take it as soon as you remember.<br>PrEP can be taken any time of the day, with or without food.<br>Taking PrEP is your responsibility.<br>PrEP is safe and effective, even when taken with hormonal contraceptives, sex hormones, or nonprescription drugs.
Starting PrEP	PrEP only reaches its effectiveness after seven days.
Stopping PrEP	You can stop PrEP 28 days after your last exposure if you are no longer at a substantial risk. Ways to lower risk include: adopting safer sexual practices, HIV positive partner in serodiscordant couple has been on effective ART for six months and is virally suppressed, leaving sex work, consistent condom use.
PrEP and alcohol or recreational drugs	There are no PrEP interactions with recreational drugs or alcohol.
No STI protection other than HIV	PrEP does not prevent any other sexually transmitted infections. Condoms used in every act of sexual intercourse provides protection against many of these infections.
No contraceptive effects	PrEP does not prevent pregnancy.

HIV testing	You need to get an HIV test before starting PrEP, or when you want to re-start PrEP after you have discontinued for a period of time.
Overall safety	PrEP is very safe. Majority of users (90 percent) will not experience any side effects.
Minor side effects	10 percent of people will experience mild side effects. These can include:<br>▪ Gastrointestinal symptoms (diarrhea and nausea, decreased appetite, abdominal cramping, and flatulence)<br>▪ Dizziness<br>▪ Headaches<br>▪ Most of those symptoms are mild and will disappear within one month. People taking PrEP should be supported and reassured.
Kidney side effects	A very small number of people will not be able to take PrEP because they have problems with their kidneys. For this reason, it is important for people taking PrEP to have a baseline creatinine test and repeat the test every six months.
Hepatitis B	You can have a blood test to see if you have hepatitis B. If the screening test is negative, you will benefit from a hep B vaccination.<br><br>If the result is positive, you can still benefit from PrEP as TDF and 3TC are recommended for treatment of hepatitis B. Further assessment can include liver function tests.<br><br>However, caution should be taken when clients discontinue PrEP as this could cause a clinical rebound of hepatitis B if they have hepatitis B associated liver disease.
PrEP during pregnancy and breast-feeding	You can use PrEP throughout pregnancy and breast-feeding.

PrEP counselling should:

- Be client-driven, based on their needs, resources, and preferences; it is not prescriptive.
- Recognize that behavior change is not easy, and human beings are not perfect.
- Focus on the identification of "small wins," more achievable "next steps" in reducing risk and/or making pill taking easier.

Examples of good counselling messages:

"You've decided to use PrEP as a way to protect yourself and that's great."

"Pill taking isn't easy and takes some practice, especially if you aren't used to taking pills."

"It's okay to not be perfect at taking your pill; it takes time. But, remember, in order for PrEP to work, you have to take your pills regularly."

"I'm here to help by working with you to figure out a way to make taking your pills easier so that you get the most protection you can."

Documentation and data management

PrEP DATA COLLECTION TOOLS

The following tools have been developed and should be used by facilities participating in PrEP demonstration projects.

Tool	Purpose	Guidance
PrEP risk assessment	To identify clients at substantial risk for acquiring HIV infection	• Tool can be used by HTS counsellors or other HCWs performing HIV testing or post-test counselling to HIV-negative clients.<br>• The tool can be used to initiate a discussion about individual risk.<br>• HCWs can indicate when clients belong to target population for PrEP (e.g., key population or vulnerable population).<br>• If the client is not identified to be at risk → leave form in screening book.<br>• If client is identified to be at risk → tear out form, refer to clinician to continue with PrEP eligibility assessment.
PrEP eligibility assessment	To ensure clients are eligible for PrEP and have no contraindications	• Tool to be used by clinicians for clients identified as being at substantial risk for HIV.<br>• Documentation of baseline laboratory results.<br>• Checklist to ensure there are no contraindications for PrEP or reasons to delay PrEP initiation.<br>• Indicate if informed consent for PrEP was obtained and client is initiated.

PrEP file	To record clinical data for client monitoring	• Every client initiating PrEP should have a PrEP file.<br>• Allocated PrEP ID (sequential number from PrEP register) to be entered on the front of the file.<br>• Client demographics should be completed.<br>• Eligibility assessment should be placed in the PrEP file.<br>• Laboratory result slips should be placed in the PrEP file.<br>• All follow-up visits should be recorded in PrEP file.<br>• Counselling checklist used and updated after each visit.
PrEP register	To monitor outcome of all PrEP clients	• Every client initiating PrEP should be entered in PrEP register.<br>• Client follow-up HIV testing and PrEP status should be recorded at 1, 3, 6, 9, 12, 15, 18 months.<br>• Register will be used to complete monthly summary forms.
PrEP monthly summary form	To monitor uptake of PrEP services at facility level	• Should be completed within five working days of new month.<br>• Will be collected by supporting partner to provide feedback to Swaziland National ART Program (SNAP).
PrEP client appointment card	To remind client of next appointment date	• Write client file/PrEP ID number on card.<br>• Write PrEP start date.<br>• Indicate follow-up visit and preferred PrEP service delivery point.

Flowchart for PrEP Implementation

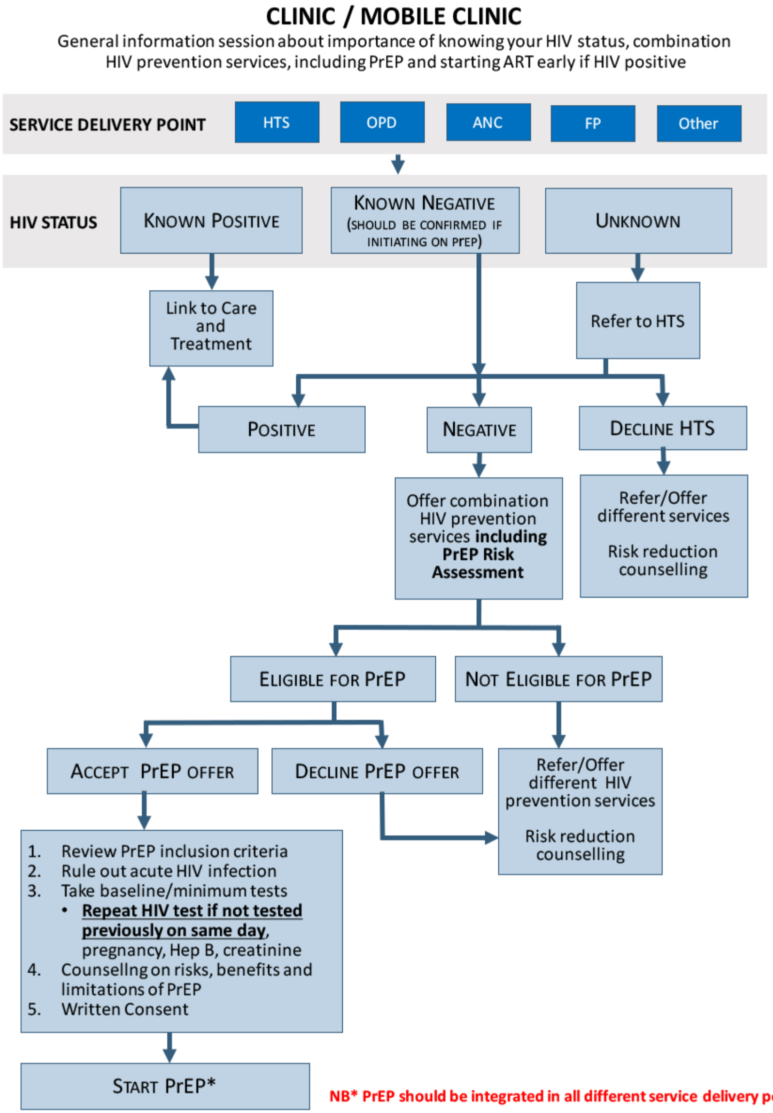

NB* PrEP should be integrated in all different service delivery points.
