# Supplementary material for: Human resource needs and costs for HIV pre-exposure prophylaxis provision in nurse-led primary care in Eswatini and opportunities for task sharing﻿
Source: Hum Resour Health. 2022 Oct 23;20:75. doi: 10.1186/s12960-022-00770-9 (PMC9590230; doi:10.1186/s12960-022-00770-9)
Supplement: Supplementary file 4 — Additional file 4. Supplement D: Time-and-motion form used for data collection in a PrEP demonstration project in Eswatini [file 12960_2022_770_MOESM4_ESM.docx]

**Supplement D:** Time-and-motion form used for data collection in a PrEP demonstration project in Eswatini


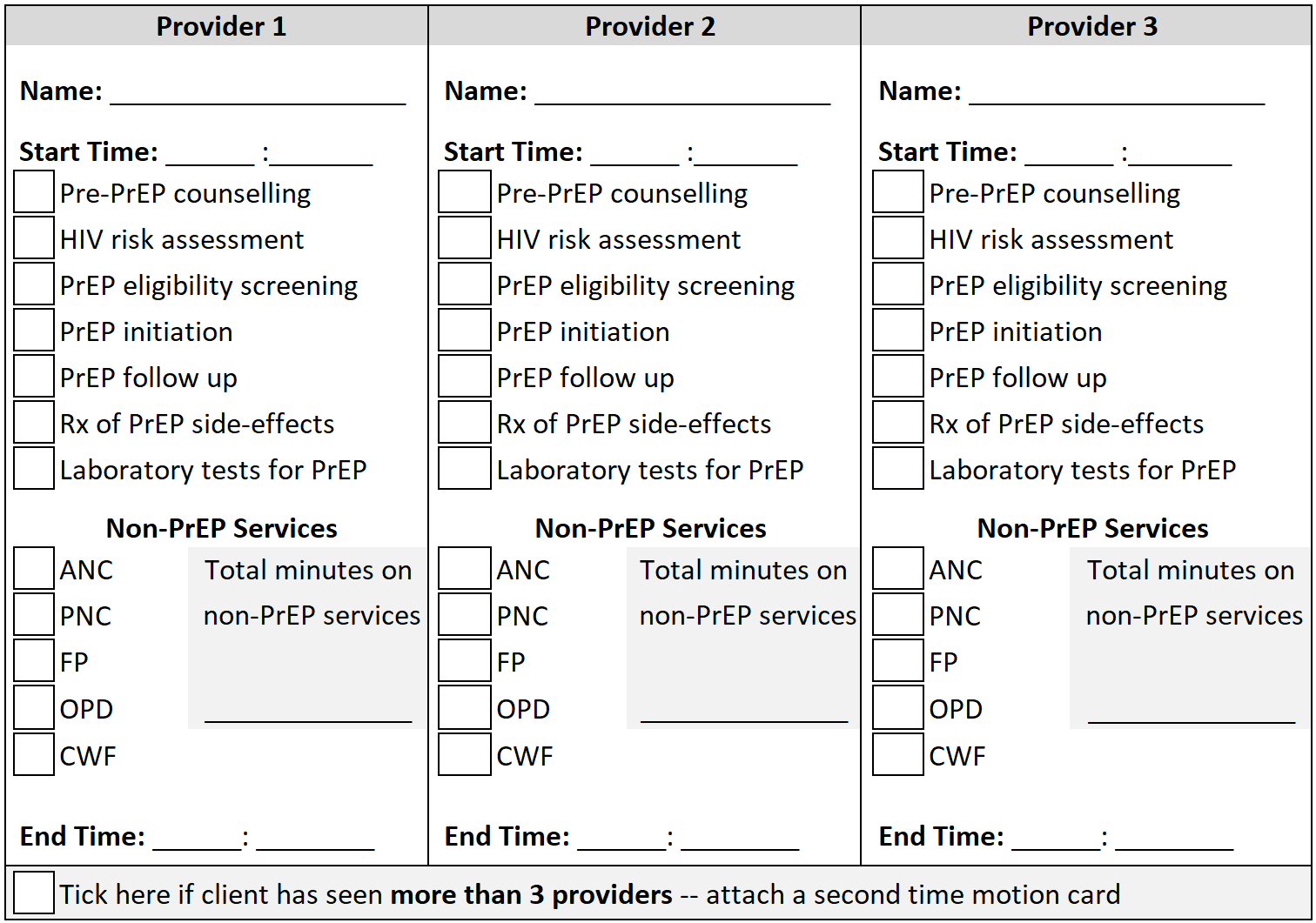


ANC = antenatal care. PNC = postnatal care. FP = family planning. OPD = outpatient department. CWF = child welfare services. The date, HIV risk assessment number, PrEP ID, facility and data collector were recorded on the backside of the time-and-motion form. One column in the time-and-motion data collection card corresponds to one client-health care worker interaction. Each client-health care worker interaction could include one or more PrEP activities and, optionally, one or more non-PrEP services. A client could receive PrEP activities from one or more health care workers and/or interact with the same health care worker more than once during the clinic visit.
